# Use of the prognostic biomarker suPAR in the emergency department improves risk stratification but has no effect on mortality: a cluster-randomized clinical trial (TRIAGE III)

**DOI:** 10.1186/s13049-018-0539-5

**Published:** 2018-08-28

**Authors:** Martin Schultz, Line Jee Hartmann Rasmussen, Malene H. Andersen, Jakob S. Stefansson, Alexander C. Falkentoft, Morten Alstrup, Andreas Sandø, Sarah L. K. Holle, Jeppe Meyer, Peter B. S. Törnkvist, Thomas Høi-Hansen, Erik Kjøller, Birgitte Nybo Jensen, Morten Lind, Lisbet Ravn, Thomas Kallemose, Theis Lange, Lars Køber, Lars Simon Rasmussen, Jesper Eugen-Olsen, Kasper Karmark Iversen

**Affiliations:** 1 Department of Cardiology, Herlev and Gentofte Hospital, University of Copenhagen, Ringvej 75, 2730 Herlev, Denmark; 2 Department of Internal medicine and Geriatrics, Herlev and Gentofte Hospital, University of Copenhagen, Ringvej 75, 2730 Herlev, Denmark; 3 Clinical Research Centre, Amager and Hvidovre Hospital, University of Copenhagen, Kettegård Alle 30, 2650 Hvidovre, Denmark; 4 Department of Anaesthesia, Centre of Head and Orthopaedics, Rigshospitalet, University of Copenhagen, Blegdamsvej 9, 2100 Copenhagen, Denmark; 5 Department of Cardiology, Rigshospitalet, University of Copenhagen, Blegdamsvej 9, 2100 Copenhagen, Denmark; 6 Department of Emergency Medicine, Bispebjerg Hospital, University of Copenhagen, Bispebjerg Bakke 23, 2400 Copenhagen, Denmark; 7 Department of Emergency Medicine, Herlev and Gentofte Hospital, University of Copenhagen, Herlev ringvej 75, 2730 Herlev, Denmark; 8 Department of Public Health, University of Copenhagen, Section of biostatistics, Øster Farimagsgade 5, 1014 Copenhagen, Denmark; 9 Center for Statistical Science, Peking University, No. 5 Yiheyuan Road Haidian District, Beijing, 100871 China

**Keywords:** Prognostic biomarkers, Risk stratification, Emergency department

## Abstract

**Background:**

Risk stratification of patients in the emergency department can be strengthened using prognostic biomarkers, but the impact on patient prognosis is unknown. The aim of the TRIAGE III trial was to investigate whether the introduction of the prognostic and nonspecific biomarker: soluble urokinase plasminogen activator receptor (suPAR) for risk stratification in the emergency department reduces mortality in acutely admitted patients.

**Methods:**

The TRIAGE III trial was a cluster-randomized interventional trial conducted at emergency departments in the Capitol Region of Denmark. Eligible hospitals were required to have an emergency department with an intake of acute medical and surgical patients and no previous access to suPAR measurement. Three emergency departments were randomized; one withdrew shortly after the trial began. The inclusion period was from January through June of 2016 consisting of twelve cluster-periods of 3-weeks alternating between intervention and control and a subsequent follow-up of ten months. Patients were allocated to the intervention if they arrived in interventional periods, where suPAR measurement was routinely analysed at arrival. In the control periods suPAR measurement was not performed. The main outcome was all-cause mortality 10 months after arrival of the last patient in the inclusion period. Secondary outcomes included 30-day mortality.

**Results:**

The trial enrolled a consecutive cohort of 16,801 acutely admitted patients; all were included in the analyses. The intervention group consisted of 6 cluster periods with 8900 patients and the control group consisted of 6 cluster periods with 7901 patients. After a median follow-up of 362 days, death occurred in 1241 patients (13.9%) in the intervention group and in 1126 patients (14.3%) in the control group.

The weighted Cox model found a hazard ratio of 0.97 (95% confidence interval, 0.89 to 1.07; *p* = 0.57). Analysis of all subgroups and of 30-day all-cause mortality showed similar results.

**Conclusions:**

The TRIAGE III trial found no effect of introducing the nonspecific and prognostic biomarker suPAR in emergency departments on short- or long-term all-cause mortality among acutely admitted patients. Further research is required to evaluate how prognostic biomarkers can be implemented in routine clinical practice.

**Trial registration:**

clinicaltrials.gov, NCT02643459. Registered 31 December 2015.

**Electronic supplementary material:**

The online version of this article (10.1186/s13049-018-0539-5) contains supplementary material, which is available to authorized users.

## Background

In emergency departments (EDs) that serve a high number of patients, delays and crowding can increase mortality [1–3]. ED patients present with all types of conditions, ranging from minor injuries to life-threatening diseases, and patient health status ranges from healthy to multimorbid. Thus, it is important to be able to distinguish between patients that can wait for treatment and those who are in need of immediate attention. Currently, many EDs use triage algorithms based upon vital signs and primary complaints to risk stratify patients.

Several studies have demonstrated that the prognostic abilities of various biomarkers in acutely admitted patients in improving risk assessment in EDs [4–9]. These biomarkers include lactate [10], copeptin [8, 9], pro-adrenomedullin [5, 6, 11], albumin [12, 13], C-reactive protein (CRP) [14, 15], and soluble urokinase plasminogen activator receptor (suPAR) [16–20]. However, none of these studies have addressed whether risk assessment that is strengthened by prognostic biomarkers improves patient outcome. This trial investigated whether using a biomarker to add prognostic information reduces mortality in ED patients.

Based upon its performance in comparable cohorts of unselected patients in EDs [16–18, 21], we used suPAR as the prognostic biomarker in this trial. suPAR is the soluble form of the cell membrane-bound protein uPAR, which is expressed mainly on immune cells, endothelial cells, and smooth muscle cells. uPAR is released during inflammation or immune activation, and the suPAR level reflects the extent of immune activation in the individual [22]. Studies have shown that the suPAR level is associated with morbidity and mortality in several acute and chronic diseases, cancer and in disease development in the general population [20]. The suPAR level is elevated across diseases, while low suPAR levels are associated with a low risk of morbidity and mortality [17], enabling an identification of patients at high and at low risk. Therefore, suPAR is applicable as a nonspecific prognostic marker and not as a diagnostic marker. In cohorts of ED patients, suPAR is associated with length of hospital stay and readmission, transfer to the intensive care unit (ICU), the presence and severity of acute and chronic conditions, and risk of death [16–18, 20, 21]. We hypothesized that availability of suPAR would improve patient prognosis.

The aim of this trial was to evaluate whether the introduction of suPAR in the emergency departments would improve risk stratification and lead to a reduction in all-cause mortality 10 months after arrival.

## Methods

### Study design and setting

The TRIAGE III trial was a cross-over, cluster-randomized, parallel-group, interventional trial. The hospitals were the units of randomization, and the patients were the units of analysis. The trial protocol was published previously [23]. The full protocol is included in Additional file 1. Using a cluster design ensured that unselected patients with different chronic and acute diseases were included in both groups as consecutive cohorts with a high inclusion rate. The trial was conducted in the Capital Region of Denmark, and eligible hospitals were required to have an ED with intake of both acute medical and surgical patients and no previous access to suPAR measurement. The inclusion criteria for the patients were age 16 years or older, acute presentation at the ED, and having blood test results (including haemoglobin, CRP, and creatinine level determination) within six hours of arrival to the ED. Patients were treated at specialized areas of the ED depending on their primary complaint. Admissions to the paediatric, obstetric, and gynaecological departments were excluded.

### Randomization

The participating EDs were randomized 1:1 to start as either intervention or control with subsequent cross over. The inclusion period consisted of twelve 3-week clusters that alternated between intervention and control periods at the two EDs. The investigators enrolled the EDs and used computer-generated numbers for randomization and allocation. Heads of the EDs provided consent before randomization.

### Intervention

In the intervention period, suPAR measurement was included as a routine test as part of the panel of blood tests that was analysed at arrival to the ED. The suPAR result was presented to the doctors on monitors and in the electronical patient journals within 2 h of blood sampling. In the control periods, the suPAR level was not measured. Blinding was not possible. Due to the nonspecific nature of suPAR, no formal intervention was defined. Instead, we provided ED doctors with an estimate of the prognosis using unadjusted mortality rates at different suPAR levels [23] and advised the doctors to incorporate the prognostic information conveyed by the suPAR level into their clinical assessment. In addition, the doctors were advised to conduct a more extensive assessment and to search for unrecognized disease in patients who had elevated suPAR levels for no obvious reason. In patients with low suPAR levels, doctors were advised to consider discharge if no other finding contradicted this. Furthermore, the ED nurses were advised to prioritise patients with high suPAR levels. Prior to the study, doctors received information regarding the prognostic abilities of suPAR, including a summary of the existing literature and pocket cards [23]. In addition, formalized teaching sessions were carried out in participating departments [23].

### Outcomes

The primary outcome was all-cause mortality assessed after April 6, 2017, ten months after inclusion of the last patient (median 12 months). The Secondary outcomes were I) all-cause mortality 30 days after arrival to the ED; II) rapid discharges (< 24 h) from the hospital; III) admissions to the medical ward (all internal medicine specialities, including admission in the “medical” area of the ED); IV) transfers to the ICU; V) new (not previously registered) cancer diagnoses at the end of follow-up; VI) the length of the hospital stay; and VII) readmission within 30 and 90 days. All outcomes were assessed after data was available in July of 2017 and prespecified in the statistical analysis plan that was published online on April 5, 2017 [24].

### Data

All residents in Denmark are registered in the Danish Civil Registration System (CRS) and have a unique personal identification number that allows follow-up through national registries. Vital status is registered in the CRS, and all patient encounters within the secondary health care system are registered in the Danish National Patient Registry (DNPR) [25–27]. We acquired data on all outcomes from the CRS and the DNPR after follow-up was complete. Data from blood tests, including the plasma suPAR level, was extracted from the hospitals laboratory databases, via the Departments of Clinical Biochemistry.

The index ED visit was defined as the first visit during the inclusion period. Readmissions was defined as a new ED visit following discharge from the ED or a hospital ward. For inclusion in the trial, patients were required to have an encounter for an acute hospital visit registered in the DNPR. The Charlson Comorbidity Index (Charlson score) was calculated using a modified SAS macro [28, 29] and based on all diagnoses in the NPR that were registered two years prior to the index ED visit [16].

### Statistical analysis

The trial was designed to have a power of 80% at a 5% level of significance and an assumption of equal cluster size to detect an absolute risk reduction in mortality of 1.5% 10 months after arrival of the last patient, with inclusion of 7340 patients in each group. Patients admitted during the intervention period without suPAR measurement remained in the intervention group for analyses according to the intention-to-treat principle. Continuous variables are described by the median value and interquartile range (IQR) and by the mean value and standard deviation (SD). Categorical variables are described by number (no.) and percentage (%). Comparing of secondary outcomes was done using Student’s t-test, Fisher’s test and Chi-square test. The statistical analysis plan is provided in Additional file 1.

### Primary outcome analyses

The main analysis of the primary outcome was performed using a weighted Cox regression model with the time since the index visit as the underlying scale. Patients who were readmitted were re-weighted at their first ED visit according to the probability of being included in the same group as their index ED visit and were otherwise censored. Subsequent readmissions did not influence weighting or censoring. Robust standard errors were employed to account for the clustering.

The following sensitivity analyses of the primary outcome were performed: I) censoring in case of readmission instead of weighting and II) a weighted analysis that excluded patients who did not receive the intervention and patients from control periods with an erroneous suPAR measurement (per protocol); III) a multivariate Cox model that was adjusted for age, sex, Charlson score, hospital, and CRP level. In addition, we performed pre-specified subgroup analyses (hospital, age < 65, age ≥ 65, groups based on discharge-diagnoses; cancer, cardiovascular disease, infections, neurological disease and surgery during admission) of the primary outcome [24].

### Secondary outcome analyses

The secondary outcome of all-cause mortality at 30 days was analysed similar to the primary outcome. The Student’s t-test was used to compare the length of stay, and the chi-square test or Fisher’s tests were used as appropriate to compare proportions. Readmissions were assessed as proportions and, additionally, with a Cox model, taking competing risks (death, readmissions) into account. Prespecified subgroup analyses were performed for the length of hospital stay, the proportion of rapid discharges within 24 h, and readmissions within 30 days [24].

### Prognostic value of suPAR

To evaluate if the prognostic value of suPAR was comparable to previous reports, we assessed the ability of suPAR to discriminate between mortality rates at 30 days and 10 months was assessed using the area under the curve (AUC) for receiver operating characteristics (ROC) curves as well as using a Cox model that was stratified by suPAR quartiles. The median suPAR levels between survivors and non-survivors were compared using the Wilcoxon rank-sum test.

### Evaluation of the intervention

To assess whether the intervention was successful and to evaluate whether the suPAR level was noticed and acted upon, we conducted an anonymous web-based questionnaire of 200 randomly selected ED doctors at the participating hospitals.

*P* < 0.05 was considered statistically significant. Statistics were performed in R version 3.2.3 (The R Foundation for Statistical Computing), and figures were created with R and GraphPad Prism, version 7.02 (GraphPad Software, Inc.). All of the analyses were stipulated in the statistical analysis plan that was published online on April 5, 2017 [24].

## Results

### Trial population

Of four eligible hospitals in the Capital Region of Denmark, one already performed suPAR measurement as a routine test and was therefore excluded, and the other three agreed to participate in the trial. One hospital withdrew from the trial shortly after the inclusion period began for internal administrative reasons. Thus, inclusion took place at two EDs at Bispebjerg Hospital and Herlev Hospital, which have annual admissions of 70,000 and 85,000, respectively. Patients were included as planned from January 11, 2016 until June 6, 2016 with a subsequent 10-month follow-up that was concluded on April 6, 2017.

**Fig. 1:**
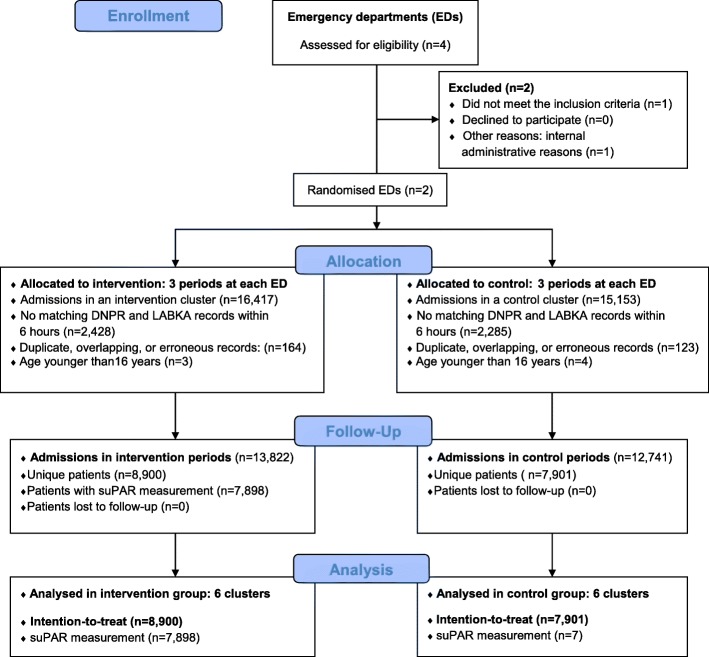
CONSORT flow diagram of the TRIAGE III trial population of patients acutely admitted. EDs: emergency departments, DNPR: National Patient Registry, LABKA: Electronical laboratory database. Full CONSORT checklist is provided in Additional file 3

Data were collected for 31,570 ED visits of 17,451 individual patients. After data management and the application of inclusion- and exclusion criteria, 26,653 ED visits of 16,801 unique patients was admitted (Fig. 1). Median age was 64 years (IQR, 45 to 77), 8864 (53%) were female. The intervention group consisted of 8900 patients compared to the control group of 7901. In the intervention group 1002 patients (11.3%) did not have a suPAR measurement available, while suPAR was erroneously measured in seven patients (0.1%) in the control group. The baseline characteristics were similar in the groups (Table 1). Comparison at the cluster level revealed small differences (Additional file 2: Table S1).

**Table 1:** Characteristics of the acutely admitted patients at index ED visit at the emergency department

	Intervention (*N* = 8900)	Control (*N* = 7901)
Hospital, number patients (%)		
Bispebjerg Hospital	3451 (38.8%)	3569 (45.2%)
Herlev Hospital	5449 (61.2%)	4332 (54.8%)
Patients		
Female sex, no. (%)	4689 (52.7%)	4175 (52.8%)
Age (years), mean (SD)	60.4 (20.8)	60.9 (20.7)
Charlson score, mean (SD)	0.7 (1.5)	0.7 (1.5)
Length of hospital stay (days), mean (SD)	4.4 (8.3)	4.5 (8.7)
In-hospital admission, no. (%)	4461 (50.1)	4031 (51.0)
Biomarkers, median (IQR)		
Albumin (g/L)	39 (35 to 43)	39 (34 to 42)
Creatinine (μmol/L)	76 (62 to 94)	75.0 (62 to 93)
CRP (mg/L)	5.0 (3.0 to 39.0)	5.0 (3.0 to 41.0)
Haemoglobin (mmol/L)	8.3 (7.5 to 9.0)	8.3 (7.6 to 9.1)
suPAR (ng/ml)	4.1 (2.9 to 6.0)	n.a.
Subgroups, diagnoses at discharge		
Cancer, no. (%)	559 (6.3%)	471 (6%)
Cardiovascular disease, no. (%)	1918 (21.6%)	1726 (21.8%)
Infections, no. (%)	1676 (18.8%)	1584 (20%)
Neurological disease, no. (%)	936 (10.5%)	864 (10.9%)
Surgery during admission, no. (%)	876 (9.8%)	787 (10%)

*CRP* C-reactive protein, *IQR* interquartile range, *SD* standard deviation, *suPAR* soluble urokinase plasminogen activator receptor

### Primary outcome

**Fig. 2:**
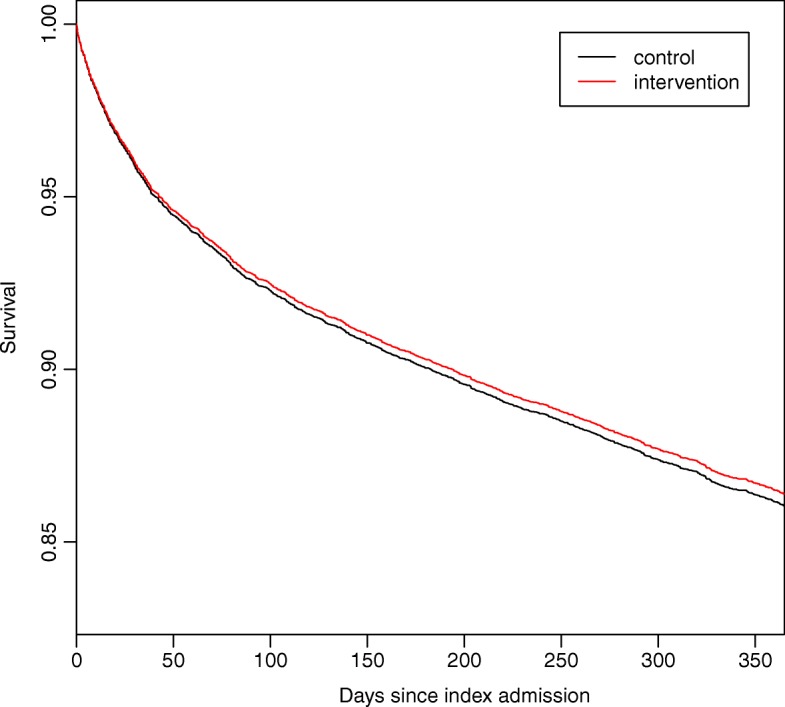
Kaplan-Meier plot displaying survival until end of follow-up. The Kaplan-Meier plot shows survival of patients acutely admitted to two emergency departments stratified by intervention period (measurement of soluble urokinase plasminogen activator receptor, suPAR) and control period (no suPAR measurement). Log-rank test: *P* = 0.61

The median follow-up in the intervention group was 362 days (IQR 325 to 397 days) and 362 (IQR 325 to 414 days). All-cause mortality as assessed at the end of follow-up occurred in 1241 patients (13.9%) in the intervention group and in 1126 patients (14.3%) in the control group. The primary weighted Cox analysis of all-cause mortality according to the intention-to-treat principle found no significant difference between the intervention group and the control group with a hazard ratio (HR) of 0.97 (95% confidence interval (CI) 0.89 to 1.07; *p* = 0.57) (Fig. 2).

**Fig. 3:**
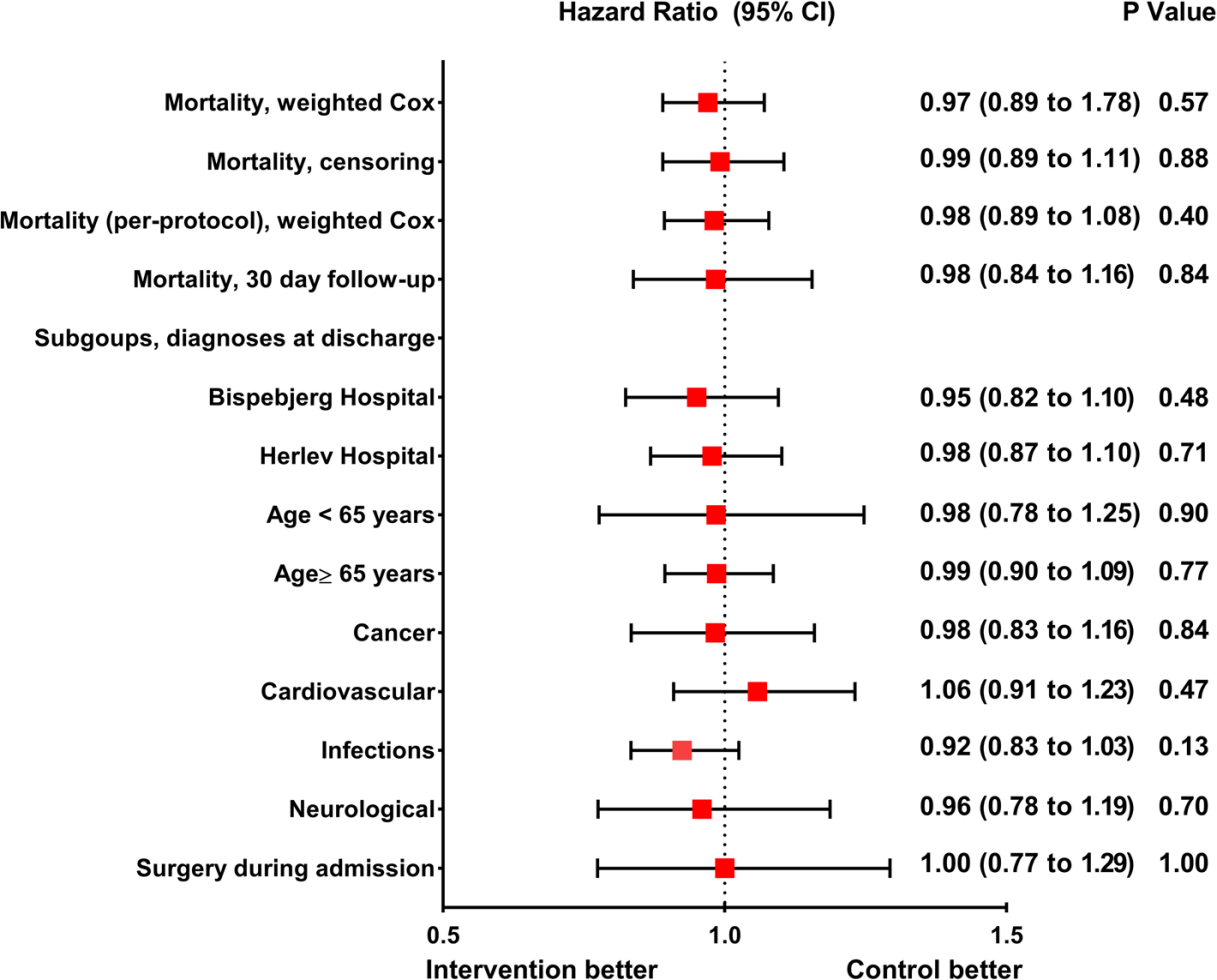
Plot of all Cox regressions from the TRIAGE III trial. Patients acutely admitted to two emergency departments were allocated to intervention (measurement of soluble urokinase plasminogen activator receptor (suPAR)) or control (no suPAR measurement). The red squares indicate point estimates and the black horizontal lines indicate 95% confidence intervals (CIs). The figure shows hazard ratios based on unadjusted weighted Cox regression models with all-cause mortality at the end of follow-up. The primary outcome of all-cause mortality assessed at the end of follow-up and sensitivity analyses (censoring and per-protocol) are included, as are the subgroups, including cluster and age. The unadjusted model with 30-day all-cause mortality as outcome is also included

None of the sensitivity and subgroup analyses, including the per-protocol and fully adjusted model showed any significant differences between the intervention and control groups (Fig. 3 and Additional file 2: Table S2).

### Secondary outcomes

Repeating the primary analysis, using all-cause mortality at 30 days showed no significant difference between groups (HR, 0.98; 95% CI, 0.84 to 1.16; *p* = 0.84). There was no significant difference between groups in the proportion of patients discharged within 24 h (intervention 49.8% vs. control 48.9%; absolute difference: 0.9%; 95% CI, − 0.62 to 2.42, *p* = 0.23). Similarly, we observed no significant difference in the mean (SD) length of hospital stay (intervention 4.39 days (8.27) vs. control 4.53 days (8.70); difference: 0.14 days; 95% CI, − 0.12 to 0.40, *p* = 0.29), nor in mean (SD) length of ED stay (intervention 4.4 h (16.5) vs. control 4.5 h (9.2), *p* = 0.61), and transfers to the ICU were equal in both groups (1.3%, *p* = 0.91). There was no difference in the frequency of newly diagnosed cancers at the end of the follow-up (intervention 8.7% vs. control 8.9%, *p* = 0.79). The proportion of admissions to the medical ward (including visits in the medical area of the ED) was significantly lower in the intervention group compared to the control group (44.3% vs. 42.0%, *p* = 0.003).

We observed a significantly higher proportion of readmissions in the intervention group at 30 days (intervention 10.3% vs. control 8.7%, *p* = 0.01), but not at 90 days (18.7% vs. 18.4%, *p* = 0.95). Cox analyses with competing risks showed the same associations: the HR for readmission risk at 30 days compared with the control group was 1.16 (95% CI, 1.05 to 1.28, *p* = 0.003) and the HR for readmission risk at 90 days was 1.02 (95% CI, 0.94 to 1.10, *p* = 0.64). The subgroup analyses are shown in Additional file 2: Table S3.

### Discriminative abilities of suPAR

**Fig. 4:**
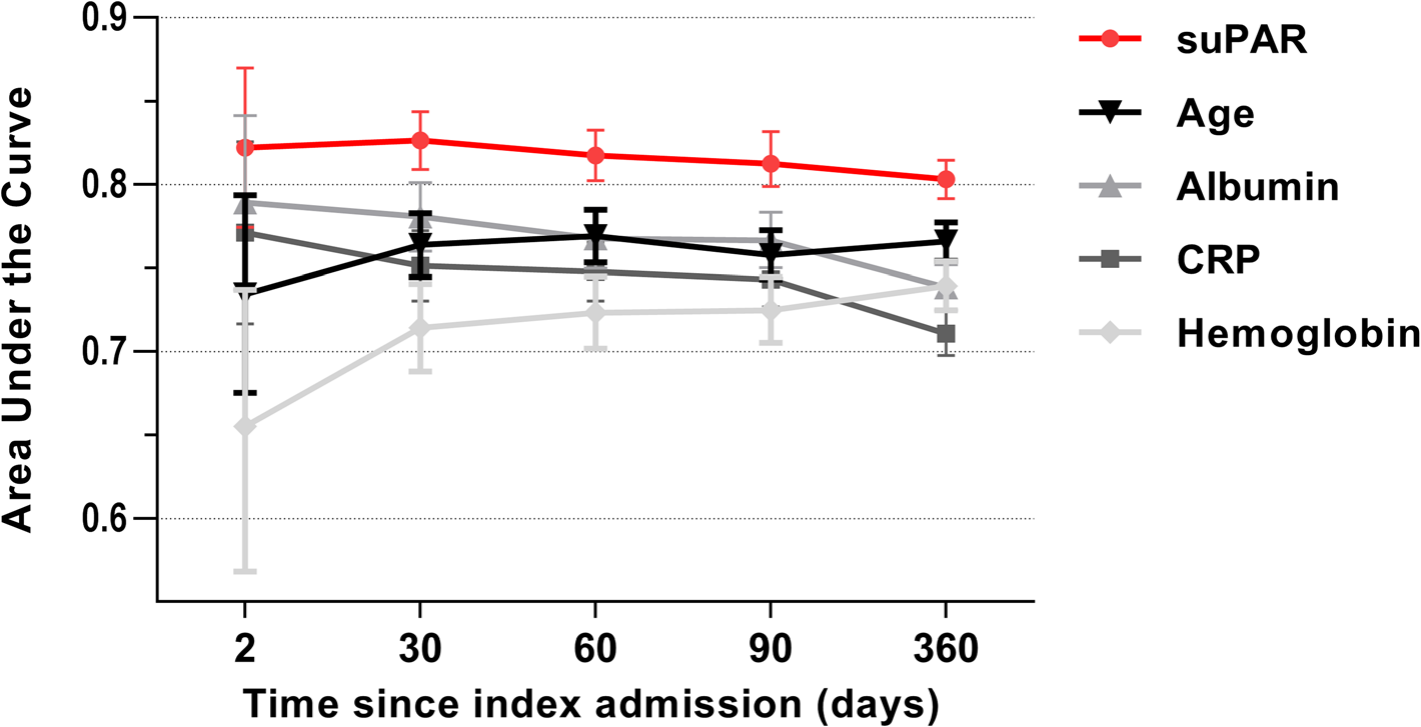
The area under the curve for mortality in patients acutely admitted. Comparison of prognostic ability of four biomarkers and age at 2 days, 30 days, 60 days, 90 days, and at the end of follow-up. suPAR vs. CRP, all time points: *P* < 0.001. suPAR vs. haemoglobin, all time points: *P* < 0.001. SuPAR vs albumin: 2 days: *P* = 0.37, suPAR vs. albumin at other time points: *P* < 0.001. CRP: C-reactive protein, suPAR: soluble urokinase plasminogen activator receptor

The median suPAR level of patients who survived was significantly lower than in patients who died during follow-up, both within 30 days (4.0 ng/ml (IQR 2.9–5.7 ng/mL) vs. 8.3 ng/ml (IQR 5.9 to 11.7 ng/mL), *p* < 0.001) and at the end of follow-up (3.8 ng/ml (IQR 2.8 to 5.3 ng/mL) vs. 6.9 ng/ml (IQR 5.1 to 10.1 ng/mL), *p* < 0.001) Stratifying patients by suPAR quartiles revealed strong discriminative abilities regarding all-cause mortality during follow-up (Additional file 2: Figure S1). The prognostic power for predicting 30-day and 10-month mortality was also high, with AUCs (95% CI) of 0.83 (0.81 to 0.84) and 0.80 (0.7 to 0.82), respectively, which were higher than for other biomarkers and age (Fig. 4).

### Results of the questionnaire

Among responders (*n* = 85), 89.4% noticed the suPAR level and 89.5% replied that they felt informed about its prognostic abilities. When asked whether suPAR influenced clinical decision making, 4.4% replied that it did in more than 20% of cases, 16.2% replied that it did in 10% to 20% of cases, while 79.4% replied that it did in less than 10% of cases or never (Additional file 2: Figure S2).

## Discussion

The TRIAGE III trial is the largest prospective trial and the first interventional trial to test the effects of introducing a prognostic biomarker in emergency medicine. We found that the introduction of the nonspecific biomarker suPAR for risk stratification in the ED had no effect on all-cause mortality.

There was no significant difference between the intervention and control groups for any of the predefined clinical outcomes or subgroups, except for a higher proportion of admissions to the medical ward in the control group and a higher risk of readmissions at 30 days in the intervention group. However, readmission risk according to group was opposite at the two hospitals, and there was no association with the risk of readmission at 90 days, indicating that this was a chance finding.

The trial was designed to investigate whether the availability of biomarker-based prognostic information improves outcome. It has previously been hypothesized, but never shown, that adding a prognostic biomarker would strengthen risk stratification and lead to improved patient outcome and flow in the ED [5, 6, 11]. In addition, early risk stratification has previously been suggested to improve patient outcome in some conditions, such as, sepsis [30–32] and myocardial infarction [33–35]. This is plausible, as patients could receive relevant treatment faster or be discharged without being exposed to the risks of hospitalization. However, our findings suggest that enhanced early risk stratification using suPAR did not provide additional information beyond clinical appearance and usual diagnostics in the current design that was translatable to interventions capable of influencing patient prognosis.

### Strengths and limitations

Strengths of the trial include the consecutively included and unselected cohort of medical and surgical patients from two large EDs, indicating high generalizability. In addition, the pragmatic cluster design of the trial ensured inclusion of a full cohort and optimal conditions to investigate the effect of the introduction of a prognostic biomarker. The doctors were provided with the blood level of suPAR, a strong prognostic biomarker, within 2 h of arrival, and our results demonstrated prognostic capabilities of suPAR similar to previous reports [17, 21]. The introduction of suPAR allowed for an enhanced early risk assessment, dividing patients into high and low risk groups.

The trial has several limitations. Biomarker-based prognostic information will only have an impact on outcomes if it is used and if it provides information that is not already obvious based on the patient’s clinical appearance or on other routine tests. Although 11.3% of suPAR measurements were missing and the response rate of the questionnaire was unsatisfactory, most respondents replied that they noticed the suPAR level, indicating that the intervention was sufficiently implemented. However, it is an important limitation to the trial that we were not able to explore the changes in clinical decision-making further or on an individual level, as our trial was designed to solely collect follow-up data from the National Registries, which does not include this information on clinical behaviour or single clinical interventions. Furthermore, the trial only included patients who had results of the routine blood tests available, thus the results are not transferable to patients with no indication for blood tests. Missing suPAR measurements in the intervention group might introduce selection bias, but the per-protocol analysis showed no effect on mortality in accordance with the ITT analyses, indicating no such bias in our data. Interventions based on the suPAR level also incorporated other clinical findings that might have made the prognostic information of suPAR unnecessary. Results from the questionnaire indicate that the suPAR level only influenced clinical decision-making in a small proportion of cases; thus, the possible effect was limited. Furthermore, due to the nonspecific nature of suPAR, there was no predefined single intervention usable for all patients and the doctors had to act differently in every case depending on the patient and the suPAR level. Doctors also had to rely on written information and on short presentations about how to interpret the suPAR level. It is possible that providing more information, holding courses, or using a longer implementation phase could maximize any potential effect. Finally, the impact of using suPAR might be clearer if we performed serial measurements, if we used a well-defined intervention based on the suPAR level, or if we assigned follow-up to patients with elevated suPAR levels.

The theoretical enhancement of biomarker-based risk stratification and subsequent improved prognosis is yet to be substantiated. The optimal approach for risk stratification would be an individually designed diagnostic strategy and clinical assessment at arrival, but this is often not feasible in a busy ED. Hence, there is still need for accurate tools for risk stratification. A single marker might not be applicable for all patients, but there could still be use for prognostic biomarkers in emergency medicine in selected conditions, in designs with clearly defined interventions, or if we focused on the negative predictive value for rapid discharge. However, this was beyond the scope of this trial, and future interventional studies are needed to evaluate how prognostic biomarkers can be implemented in routine clinical practice.

## Conclusion

The introduction of the nonspecific and prognostic biomarker suPAR in EDs did not affect short- or long-term all-cause mortality among unselected acutely admitted patients. Further research is required to evaluate how prognostic biomarkers can be implemented in routine clinical practice.

## Additional files

Additional file 1: Protocol and Statistical Analysis Plan, contains original and final protocols as well as the final Statistical Analysis Plan for the TRIAGE III trial. (DOCX 128 kb)

Additional file 2: Appendix contains additional and supporting tables and figures for the manuscript. (DOCX 256 kb)

Additional file 3: CONSORT checklist, documentation that the TRIAGE III trial follows the CONSORT criteria. (DOCX 29 kb)

## Abbreviations

CRP: C-reactive protein
ED: Emergency department
ICU: Intensive care unit
suPAR: soluble urokinase plasminogen activator receptor
