# Supplementary material for: Use of the prognostic biomarker suPAR in the emergency department improves risk stratification but has no effect on mortality: a cluster-randomized clinical trial (TRIAGE III)
Source: Scand J Trauma Resusc Emerg Med. 2018 Aug 28;26:69. doi: 10.1186/s13049-018-0539-5 (PMC6114851; doi:10.1186/s13049-018-0539-5)
Supplement: Supplementary file 1 — Protocol and Statistical Analysis Plan, contains original and final protocols as well as the final Statistical Analysis Plan for the TRIAGE III trial. (DOCX 128 kb) [file 13049_2018_539_MOESM1_ESM.docx]

**Protocol and Statistical analysis Plan**

**TRIAGE III trial**

All documents included are from the TRIAGE III trial.

**Table of contents**

[Original protocol (In Danish) 3](#_Toc503526478)

[Original Protocol (translated from Danish) 12](#_Toc503526479)

[Final Protocol (in Danish) 21](#_Toc503526480)

[Final Protocol (translated from Danish) 29](#_Toc503526481)

[Original Statistical analysis plan 37](#_Toc503526482)

[Final Statistical analysis plan 39](#_Toc503526483)

[Summary of changes to the protocol 45](#_Toc503526484)

[Summary of changes to the Statistical Analysis Plan 46](#_Toc503526485)

[Summary of changes at clinicaltrials.gov: 47](#_Toc503526486)

# Original protocol (In Danish)

**Betydningen af suPAR-måling i akutmodtagelser**

**(TRIAGE III)**

Teknisk protokol, V1

15. Sep 2015

**Generelle oplysninger**

**Styregruppe**

Seniorforsker, phd Jesper Eugen-Olsen, Hvidovre Hospital.

Professor, dr.med Lars S Rasmussen, Rigshospitalet.

Professor, dr.med Lars Køber, Rigshospitalet.

Overlæge, dr.med Kasper Iversen, Herlev Hospital

Overlæge dr.med Erik Kjøller, Herlev Hospital

Ledende overlæge Lisbet Ravn, Herlev Hospital

Ledende overlæge Birgitte Nybo Jensen, Bispebjerg Hospital

**Lokalisation**

Projektet udføres på Herlev Hospital, Bispebjerg Hospital og Nordsjælland Hospital

**Tidsplan**

Planlagt projektstart er 11. januar 2016 og ventes afsluttet 16. Maj 2015 kl 08.00

**Baggrund**

Risikovurdering er central ved modtagelse og vurderingen af akut indlagte patienter skal sikre, at de mest akut syge bliver behandlet først og observeres tættest. Risikovurdering foretages i dag ved hjælp af systematiseret triage. Systematiseret triage foregår typisk ved en gennemgang af patientens primære symptom samt deres vitale parametre. Både symptom og vitale parametre tillægges en numerisk værdi ud fra et scoringssystem, bestemt af triagemodellen. På denne måde opdeles patienterne i kategorier, som vha. farver eller tal signalerer, hvor hurtigt de skal tilses, samt hvor tæt de skal observeres^1^.

Gennem de seneste år er der kommet tiltagende fokus på, om man kan forbedre risikovurderingen i hospitalernes modtagelser ved at addere resultater fra biomarkørmålinger til den eksisterende triage. Det ser ud som om, at en sådan kombineret strategi er brugbar både ved specifikke diagnoser^2-4^ og mere generelt^5-7^. Hvilke biomarkører der skal bruges, og om det rent faktisk gør en forskel for patienterne, er der indtil videre ingen data på.

Soluble urokinase plasminogen activating receptor (suPAR) er den opløselige form af urokinase-type plasminogen activator receptor (uPAR) og primært udtrykt på immunceller som neutrofile leukocytter, aktiverede T-celler og makrofager^8^. Studier af suPAR har vist, at proteinet er stærkt associeret med mortalitet og morbiditet i en lang række specifikke sygdomme (sepsis, leversygdom, hjertesygdom, lungesygdom)^9-19^, generelt ved indlagte patienter^20,21^ og i den generelle befolkning^22,23^. Derudover har suPAR vist sig at kunne identificere patienter med manifest eller med høj risiko for at udvikle cancer^24,25^

Det ser således ud til, at suPAR er helt uspecifik og derfor ikke kan bruges til at diagnosticere nogen bestemt sygdom, men udelukkende til at identificere lav- og høj-risikopatienter generelt. Dermed kunne suPAR potentielt være en god markør i akutmodtagelser til at identificere risikopatienter, der kræver tættere observation, hurtigere behandling og/eller videre udredning. På den anden side ser det også ud til at patienter med lav suPAR har en overordentlig god prognose, og suPAR vil derfor potentielt også kunne identificere patienter, der hurtigt kan udskrives. Enkelte steder (eksempelvis Hvidovre Hospital) har man allerede indført suPAR som en rutinemarkør, der tages på alle indlagte patienter i akutmodtagelsen.

Selvom det er vist, at suPAR både kan identificere høj- og lavrisiko-patienter, er det aldrig vist, at man kan bruge denne viden til at forebygge dødsfald eller alvorlige komplikationer og ej heller om det kan forbedre patientflowet på hospitalet.

**Formål**

At undersøge om måling af suPAR og information herom til læger, der arbejder i akutmodtagelsen, kan forebygge dødsfald eller alvorlige komplikationer samt forbedre patientflow hos akut indlagte medicinske og kirurgiske patienter.

**Metode**

Studiet er et åbent clusterrandomiseret studie af betydningen af suPAR-måling i akutmodtagelsen.

**Målepunkter**

*Primære endepunkt*

- Død inden for 12 måneder af alle årsager

*Sekundære endepunkter*

- Død inden for 1 måned
- Død inden for 3 mdr
- Død inden for 2 år
- Antal patienter udskrevet indenfor 24 timer
- Indlæggelser på medicinske afdelinger indenfor 30 dage
- Indlæggelser på intensivafdeling indenfor 30 dage
- Cancerdiagnoser inden for 3 måneder
- Cancerdiagnoser fra 3 – 24 måneder
- Indlæggelsesvarighed
- Akutte genindlæggelser inden for 30 dage

**Patienter**

suPAR indføres som rutinemarkør på alle indlagte patienter i 2 x 21 dages perioder på 3 matrikler i akutmodtagelsen på henholdsvis Bispebjerg, Nordsjælland og Herlev Hospital (Tabel 1). De resterende 4 x 21 dages perioder i studieperioden fungerer som kontrolperioder.

**Måling af suPAR**

Materiale til måling af suPAR tages fra i forbindelse med rutine-blodprøvetagning og medfører hverken ekstra stik til patienter eller udtagning af en større mængde blod fra patienterne end vanligt.

suPAR måles på EDTA-plasma fra overskydende materiale fra rutineblodprøverne. Analysen udføres med suPARnostic^®^ Quick Triage stick (QT stick) på Klinisk Biokemisk Afdeling. Der udtages 10 mikroliter (µl) EDTA-plasma fra rutine-blodprøverne. Disse 10 µl overføres til et præfabrikeret rør, der indeholder 100 µl running buffer. Herfra pipetteres 60 µl på QT sticken, som sættes i analyseapparatet, og der trykkes start. Efter 20 minutter aflæses resultatet automatisk og rapporteres på den tilkoblede PC. Den benyttede QT stick smides ud, og apparatet er klar til ny måling. Der opstilles 4 analyseapparater, således at 4 analyser kan køres sideløbende, og op til 12 prøver analyseres i timen. Så snart resultatet på suPAR foreligger på den til analyseapparatet tilkoblede PC, vil svaret blive skrevet ind i de elektroniske blodprøve- og journalsystemer LABKA og OPUS. Det tilstræbes, at alle suPAR-analysesvar er indskrevet i LABKA/OPUS hurtigst muligt og max 2 timer efter blodprøvetagning.

Afpippetering og analyse af suPAR foretages af studiepersonale og vil ikke medføre ekstra arbejde for de implicerede hospitaler.

**Information til hospitalets læger om suPAR**

Forud for studieperioden vil læger på de implicerede hospitaler blive undervist i den prognostiske betydning af suPAR både i forhold til specifikke diagnoser og i forhold til uselekterede patienter. Undervisningen vil foregå på de enkelte afdelinger på sygehuset. Derudover vil alle læger få tilsendt et resume over de data, der forligger om suPAR’s prognostiske betydning, og alle læger får derudover udleveret lommekort, der beskriver risiko for 30- og 90-dages mortalitet ved forskellige niveauer af suPAR, baseret på data fra Hvidovre Hospital (Figur 1).

**Dataopsamling**

Resultater fra suPAR-måling samt øvrige biokemiske målinger vil være registreret i LABKA-databasen, og data vil blive trukket herfra.

Information om primært og sekundære endepunkter indhentes fra centrale registre.

**Styrkeberegning**

I et tidligere endnu upubliceret studie af patienter indlagt i akutmodtagelsen på Nordsjælland Hospital var dødeligheden inden for 3 måneder 7,5 %. Vores hypotese er, at vi kan reducere mortaliteten med 1 % ved at måle suPAR på alle indlagte patienter. Ved et signifikansniveau på 5 % og en power på 80 % skal man, såfremt grupperne er lige store, bruge en stikprøvestørrelse på 10.042 personer i hver gruppe for at vise denne forskel. Da ratio mellem interventionsgruppen og kontrolgruppen er 1: 2 ændres gruppestørrelserne til 7665 i interventionsgruppen og 15.329 i kontrolgruppen.

**Tidsplan**

Studiet vil starte klokken 8:00 den 11/1 2016 og vil være færdigt klokken 8:00 den 16/5 2016.

Opgørelse af data til det primære endepunkt forventes færdiggjort 1/6 2017. Første udkast til den første artikel vil være færdigt inden udgangen af 2017.

Baseret på tal fra de 3 akutmodtagelser indlægges der ca. 240 patienter dagligt på de 3 hospitaler tilsammen. I studieperioden vil der således kunne opsamles data på 10.080 patienter i den aktive arm og 20.160 patienter i kontrolarmen. Dette vil være tilstrækkeligt til at undersøge den primære hypotese.

**Tilladelser**

Studiet er ikke anmeldelsespligtigt til videnskabsetisk komité (jr. nummer FSP-15003590). Studiet vil blive anmeldt til Sundhedsstyrelsen, Datatilsynet samt registreret på clinicaltrials.gov.

**Økonomi**

Udgifter i forbindelse med studiet er finansieret af firmaet ViroGates A/S. Der vil sammen med ViroGates A/S blive søgt ekstern finansiering til dele af eller hele projektet.

**Referencer**

1. Farrohknia N, Castren M, Ehrenberg A, et al. Emergency department triage scales and their components: a systematic review of the scientific evidence. Scandinavian journal of trauma, resuscitation and emergency medicine 2011;19:42.

2. Albrich WC, Ruegger K, Dusemund F, et al. Biomarker-enhanced triage in respiratory infections: a proof-of-concept feasibility trial. The European respiratory journal 2013;42:1064-75.

3. Sibon I, Rouanet F, Meissner W, Orgogozo JM. Use of the Triage Stroke Panel in a neurologic emergency service. The American journal of emergency medicine 2009;27:558-62.

4. Vanni S, Polidori G, Pepe G, et al. Use of biomarkers in triage of patients with suspected stroke. The Journal of emergency medicine 2011;40:499-505.

5. Gans SL, Atema JJ, Stoker J, Toorenvliet BR, Laurell H, Boermeester MA. C-reactive protein and white blood cell count as triage test between urgent and nonurgent conditions in 2961 patients with acute abdominal pain. Medicine 2015;94:e569.

6. Herlitz J, Svensson L. The value of biochemical markers for risk stratification prior to hospital admission in acute chest pain. Acute cardiac care 2008;10:197-204.

7. Seymour CW, Cooke CR, Wang Z, et al. Improving risk classification of critical illness with biomarkers: a simulation study. Journal of critical care 2013;28:541-8.

8. Huai Q, Mazar AP, Kuo A, et al. Structure of human urokinase plasminogen activator in complex with its receptor. Science (New York, NY) 2006;311:656-9.

9. Borne Y, Persson M, Melander O, Smith JG, Engstrom G. Increased plasma level of soluble urokinase plasminogen activator receptor is associated with incidence of heart failure but not atrial fibrillation. European journal of heart failure 2014;16:377-83.

10. Eapen DJ, Manocha P, Ghasemzedah N, et al. Soluble urokinase plasminogen activator receptor level is an independent predictor of the presence and severity of coronary artery disease and of future adverse events. Journal of the American Heart Association 2014;3:e001118.

11. Gumus A, Altintas N, Cinarka H, et al. Soluble urokinase-type plasminogen activator receptor is a novel biomarker predicting acute exacerbation in COPD. International journal of chronic obstructive pulmonary disease 2015;10:357-65.

12. Lyngbaek S, Andersson C, Marott JL, et al. Soluble urokinase plasminogen activator receptor for risk prediction in patients admitted with acute chest pain. Clinical chemistry 2013;59:1621-9.

13. Lyngbaek S, Marott JL, Moller DV, et al. Usefulness of soluble urokinase plasminogen activator receptor to predict repeat myocardial infarction and mortality in patients with ST-segment elevation myocardial infarction undergoing primary percutaneous intervention. The American journal of cardiology 2012;110:1756-63.

14. Molkanen T, Ruotsalainen E, Thorball CW, Jarvinen A. Elevated soluble urokinase plasminogen activator receptor (suPAR) predicts mortality in Staphylococcus aureus bacteremia. European journal of clinical microbiology & infectious diseases : official publication of the European Society of Clinical Microbiology 2011;30:1417-24.

15. Persson M, Ostling G, Smith G, et al. Soluble urokinase plasminogen activator receptor: a risk factor for carotid plaque, stroke, and coronary artery disease. Stroke; a journal of cerebral circulation 2014;45:18-23.

16. Raggam RB, Wagner J, Pruller F, et al. Soluble urokinase plasminogen activator receptor predicts mortality in patients with systemic inflammatory response syndrome. Journal of internal medicine 2014;276:651-8.

17. Reichsoellner M, Raggam RB, Wagner J, Krause R, Hoenigl M. Clinical evaluation of multiple inflammation biomarkers for diagnosis and prognosis for patients with systemic inflammatory response syndrome. Journal of clinical microbiology 2014;52:4063-6.

18. Suberviola B, Castellanos-Ortega A, Ruiz Ruiz A, Lopez-Hoyos M, Santibanez M. Hospital mortality prognostication in sepsis using the new biomarkers suPAR and proADM in a single determination on ICU admission. Intensive care medicine 2013;39:1945-52.

19. Zimmermann HW, Koch A, Seidler S, Trautwein C, Tacke F. Circulating soluble urokinase plasminogen activator is elevated in patients with chronic liver disease, discriminates stage and aetiology of cirrhosis and predicts prognosis. Liver international : official journal of the International Association for the Study of the Liver 2012;32:500-9.

20. Donadello K, Scolletta S, Taccone FS, et al. Soluble urokinase-type plasminogen activator receptor as a prognostic biomarker in critically ill patients. Journal of critical care 2014;29:144-9.

21. Haupt TH, Petersen J, Ellekilde G, et al. Plasma suPAR levels are associated with mortality, admission time, and Charlson Comorbidity Index in the acutely admitted medical patient: a prospective observational study. Critical care (London, England) 2012;16:R130.

22. Eugen-Olsen J, Andersen O, Linneberg A, et al. Circulating soluble urokinase plasminogen activator receptor predicts cancer, cardiovascular disease, diabetes and mortality in the general population. Journal of internal medicine 2010;268:296-308.

23. Lyngbaek S, Marott JL, Sehestedt T, et al. Cardiovascular risk prediction in the general population with use of suPAR, CRP, and Framingham Risk Score. International journal of cardiology 2013;167:2904-11.

24. Langkilde A, Hansen TW, Ladelund S, et al. Increased plasma soluble uPAR level is a risk marker of respiratory cancer in initially cancer-free individuals. Cancer epidemiology, biomarkers & prevention : a publication of the American Association for Cancer Research, cosponsored by the American Society of Preventive Oncology 2011;20:609-18.

25. Chounta A, Ellinas C, Tzanetakou V, et al. Serum soluble urokinase plasminogen activator receptor as a screening test for the early diagnosis of hepatocellular carcinoma. Liver international : official journal of the International Association for the Study of the Liver 2015;35:601-7.

**Tabel 1**

| Hospital | Periode 1 | Periode 2 | Periode 3 | Periode 4 | Periode 5 | Periode 6 |
| --- | --- | --- | --- | --- | --- | --- |
| Herlev | + suPAR | Kontrol | Kontrol | + suPAR | Kontrol | Kontrol |
| Bispebjerg | Kontrol | + suPAR | Kontrol | Kontrol | + suPAR | + Kontrol |
| Nordsjælland | Kontrol | Kontrol | + suPAR | Kontrol | Kontrol | + suPAR |

**Figur 1:**

**suPAR og risiko for dødelighed hos akutmedicinske patienter**

**Patienter < 70 år:**

| **Mortalitetsrisiko (% (95%CI)) i henhold til suPAR-værdier hos patienter <70 år** | | |
| --- | --- | --- |
| **suPAR (ng/ml)** | **30 dage** | **90 dage** |
| **Alle (n=2652)** | **1,5% (1,1-2,0)** | **2,9% (2,2-3,6)** |
| 0-3 (n=1705) | **0,3 %** (0,1-0,7; n=5) | **0,8 %** (0,5-1,4; n=14) |
| 3-6 (n=744) | **1,6 %** (0,8-2,8; n=12) | **3,6 %** (2,4-5,2; n=27) |
| 6-9 (n=127) | **7,1 %** (3,3-13,0; n=9) | **11,8 %** (6,8-18,7; n=15) |
| >9 (n=76) | **19,7 %** (11,5-30,5; n=15) | **27,6 %** (18,0-39,1; n=21) |

**Eksempler:**

En mand/kvinde på 63 år med suPAR på 2,7 ng/ml har en risiko for 30 dages mortalitet på 0,3%, hvilket er fem gange mindre end den gennemsnitlige dødelighed (1.5%) for personer under 70 år.

En mand/kvinde på 63 år med suPAR på 7,7 ng/ml har en risiko for 30 dages mortalitet på 7,1%, hvilket er fem gange højere end den gennemsnitlige dødelighed (1.5%) for personer under 70 år.

**Patienter > 70 år:**

| **Mortalitetsrisko (% (95% CI)) i henhold til suPAR-værdier hos patienter >70 år** | | |
| --- | --- | --- |
| **suPAR ng/ml** | **30 dage** | **90 dage** |
| **Alle (n=1691)** | **10,8% (9,3-12,3)** | **18,3% (16,4-20,1)** |
| 0-3 (n=339) | **2,7 %** (1,2-5,0; n=9) | **3,5 %** (1,8-6,1; n=12) |
| 3-6 (n=913) | **7,1 %** (5,5-9,0; n=65) | **13,7 %** (11,5-16,1; n=125) |
| 6-9 (n=293) | **19,8 %** (15,4-24,8; n=58) | **32,8 %** (27,4-38,5; n=96) |
| >9 (n=146) | **34,9 %** (27,2-43,3; n=51) | **52,1 %** (43,6-60,4; n=76) |

**Eksempler**:

En mand/kvinde på 75 år med suPAR på 2.7 ng/ml har en risiko for 30 dages mortalitet på 2.7%, hvilket er fire gange mindre end den gennemsnitlige dødelighed (10.8%) for personer over 70 år.

En mand/kvinde på 75 år med suPAR på 7.7 ng/ml har en risiko for 30 dages mortalitet på 19.8%, hvilket er ca. dobbelt så høj risiko end den gennemsnitlige dødelighed (10.8%) personer over 70 år.

Kilde: Data er beregnet ud fra samtlige akutmedicinske patienter (n=4343) i Akutmodtagelsen, Hvidovre Hospital, i perioden 18. november 2013 – 31. marts 2014.

# Original Protocol (translated from Danish)

**The impact of suPAR measurement**

**in emergency departments**

**(TRIAGE III)**

Technical protocol V.1

September 15 2015 /MSC

**General information**

**Steering committee:**

Senior researcher, PhD Jesper Eugen-Olsen, Hvidovre Hospital

Professor, DMSc. Lars S Rasmussen, Rigshospitalet

Professor, DMSc. Lars Køber, Rigshospitalet

M.D., DMSc. Kasper Iversen, Herlev Hospital

M.D., DMSc. Erik Kjøller, Herlev Hospital

Chief Physician Lisbet Ravn, Herlev Hospital

Chief Physician Birgitte Nybo Jensen, Bispebjerg Hospital

**Location**

The study will take place at Herlev Hospital, Bispebjerg Hospital and Hillerød Hospital.

**Time plan**

Planned start of inclusion is on January 11 2016 at 08:00 and is expected to be concluded on May 16 2016 at 08:00.

**Background**

Risk assessment is a central concept at admittance of acutely admitted patients to ensure that the sickest patients are treated first and observed the closest. Today, systematized risk assessment is being performed with triage algorithms. According to the primary complaint and vitals sign of the patient an aggregated score is determined by the triage algorithm. Patients are then stratified in groups of acuity that signals how fast they should be assessed by doctors and the frequency of observation^1^. In recent years it has been suggested that the risk assessment in the emergency departments can be strengthened by adding results from blood-based biomarkers to the triage process. A combined strategy looks viable both in specific diagnoses^2-4^ and in general^5-7^. However, there is no data to support whether this strategy makes a difference to the patients or which biomarkers that should be used.

Soluble urokinase plasminogen activating receptor (suPAR) is the soluble form of the urokinase-type plasminogen activator receptor (uPAR), which primary is expressed on the surface of cells in the immune system (Neutrophiles cells, activated T-cells and macrophages)^8^. Studies have shown that suPAR is strongly associated with mortality and morbidity in a wide range of specific diseases (sepsis, hepatic-, cardiac- and lung-diseases) ^9-19^, among admitted patients^20,21^ and in the general population^22,23^. In addition suPAR have been shown to identify patients at low and high risk of developing cancer^24,25^. It appears that suPAR is a nonspecific marker of outcome and not suited as a diagnostic tool but solely to identify patients at low and high risk.

Thus, suPAR could potentially be a good marker in the emergency department to identify risk-patients requiring close observation, fast treatment and further investigation. On the other hand, it appears that patients with low suPAR level have a very good prognosis and are potentially suitable for rapid discharge. In few places (e.g. Hvidovre Hospital) suPAR have already been implemented as a routine analysis of all acutely admitted medical patients.

Although it has been shown that suPAR is suitable for identification of patients at high and low risk, it has never been shown if this information can be used to prevent deaths or serious complications, nor if it can improve patient flow in the emergency department (ED).

**Objective**

To assess if measurement of the suPAR level and information to the attending ED doctors can prevent serious complications and deaths among acutely admitted medical- and surgical patients and improve flow in the emergency department.

**Methods**

Triage III is an open, cluster-randomized, interventional study on the impact of introducing suPAR measurement in the emergency department.

**Outcomes**

*Primary outcome:*

- All-cause mortality within 12 months

*Secondary outcomes:*

- All-cause mortality at one month, three months and two years of follow-up
- Number of patients discharged within 24 hours
- Number of admissions at the medical wards within 30 days
- Number of transfers to the intensive care unit within 30 days
- New cancer diagnoses assessed three months after admission
- New cancer diagnoses assessed 24 months after admission
- Length of stay
- Number of readmissions within 30 days

**Patients:**

suPAR will be introduced as a routine analysis on all acutely admitted patients in two 21-day periods at the emergency departments at Herlev, Hillerød and Bispebjerg hospital. The remaining four periods of 21 will serve as control periods (Table 1).

**Measurement of suPAR**

Plasma for suPAR measurement will be obtained in connection with routine blood test and will not result in additional discomfort for the patients.

The suPAR level is measured on excess EDTA-plasma from the routine blood tests. The analysis is done with the suPARnostic^®^ Quick Triage stick (QT stick). From routine blood-tests 10 microliter (µl) EDTA-plasma is extracted and transferred to a prefabricated test tube containing 100 µl ”running buffer”. From the mixture, 60 µl is pipetted to the QT stick which is inserted in the analyzer. The suPAR level is automatically reported on the connected PC after 20 minutes. During the inclusion period it is possible to analyze four tests simultaneously at a minimum of twelve tests an hour. When the test result is available it will be reported to the electronic Laboratory system ”LABKA” and electronical hospital records (“OPUS”) as soon as possible and within two hours. Measurement of suPAR is done by study staff, which is present 24 hours every day during inclusion.

**Information to attending doctors regarding the suPAR level**

Prior to the study period, doctors will receive written information regarding the prognostic value of suPAR in general and with regard to specific diagnoses. In addition, all participating departments will be offered formalized teaching sessions. Doctors will receive a review of the published literature and pocket cards describing risk of 30- and 90-day mortality depending on the suPAR level based on data from Hvidovre Hospital and Hillerød hospital (Figure 1).

**Data**

SuPAR levels as well as all other biochemical test are registered in the LABKA database and can be extracted. Data on all outcomes will be obtained from the central Danish registries.

**Power calculation**

In a previous unpublished study of patients acutely admitted to the ED at Copenhagen University Hospital North Zealand, Denmark, 3-month mortality was 7.5 %.

Our main hypothesis is that we can reduce all-cause mortality with an absolute of 1% by measuring suPAR on admitted patients. Using a 5 % level of significance, a power of 80 %, and an assumption of equal group size we will need a sample of 10,042 patients in each randomization group to detect this difference. Ratio between the intervention group and the control group is 1:2, equal to 7,665 patients in the intervention group and 15,329 in the control group.

**Timeline**

The trial will start on January 11 2016 at 08:00 and be concluded on May 16 2016 at 08:00. Data on the primary outcome can be assessed at June 1 2017. First draft of the manuscript is expected at the end of 2017.

Based upon numbers from the three emergency departments, approximately 240 patients are admitted on a daily basis. During the inclusion period it will be possible to collect data on 10,080 patients in the intervention group and 20,160 patients in the control periods. This will be sufficient to assess the primary hypothesis.

**Ethics and approvals**

The trial was presented to the Regional Ethics Committee, who decided that no formal approval was needed in accordance with Danish law (Jr. Nr.: FSP-15003590). The trial will be registered at the Danish Data Protection Agency, the Danish Patient Safety Authority, and at clinicaltrials.org.

**Economy**

Expenses related to the study are financed by ViroGates A / S. Together with ViroGates external funding will be sought for parts of or the entire project.

**References**

1. Farrohknia N, Castren M, Ehrenberg A, et al. Emergency department triage scales and their components: a systematic review of the scientific evidence. Scandinavian journal of trauma, resuscitation and emergency medicine 2011;19:42.

2. Albrich WC, Ruegger K, Dusemund F, et al. Biomarker-enhanced triage in respiratory infections: a proof-of-concept feasibility trial. The European respiratory journal 2013;42:1064-75.

3. Sibon I, Rouanet F, Meissner W, Orgogozo JM. Use of the Triage Stroke Panel in a neurologic emergency service. The American journal of emergency medicine 2009;27:558-62.

4. Vanni S, Polidori G, Pepe G, et al. Use of biomarkers in triage of patients with suspected stroke. The Journal of emergency medicine 2011;40:499-505.

5. Gans SL, Atema JJ, Stoker J, Toorenvliet BR, Laurell H, Boermeester MA. C-reactive protein and white blood cell count as triage test between urgent and nonurgent conditions in 2961 patients with acute abdominal pain. Medicine 2015;94:e569.

6. Herlitz J, Svensson L. The value of biochemical markers for risk stratification prior to hospital admission in acute chest pain. Acute cardiac care 2008;10:197-204.

7. Seymour CW, Cooke CR, Wang Z, et al. Improving risk classification of critical illness with biomarkers: a simulation study. Journal of critical care 2013;28:541-8.

8. Huai Q, Mazar AP, Kuo A, et al. Structure of human urokinase plasminogen activator in complex with its receptor. Science (New York, NY) 2006;311:656-9.

9. Borne Y, Persson M, Melander O, Smith JG, Engstrom G. Increased plasma level of soluble urokinase plasminogen activator receptor is associated with incidence of heart failure but not atrial fibrillation. European journal of heart failure 2014;16:377-83.

10. Eapen DJ, Manocha P, Ghasemzedah N, et al. Soluble urokinase plasminogen activator receptor level is an Independent predictor of the presence and severity of coronary artery disease and of future adverse events. Journal of the American Heart Association 2014;3:e001118.

11. Gumus A, Altintas N, Cinarka H, et al. Soluble urokinase-type plasminogen activator receptor is a novel biomarker predicting acute exacerbation in COPD. International journal of chronic obstructive pulmonary disease 2015;10:357-65.

12. Lyngbaek S, Andersson C, Marott JL, et al. Soluble urokinase plasminogen activator receptor for risk prediction in patients admitted with acute chest pain. Clinical chemistry 2013;59:1621-9.

13. Lyngbaek S, Marott JL, Moller DV, et al. Usefulness of soluble urokinase plasminogen activator receptor to predict

repeat myocardial infarction and mortality in patients with ST-segment elevation myocardial infarction undergoing primary percutaneous intervention. The American journal of cardiology 2012;110:1756-63.

14. Molkanen T, Ruotsalainen E, Thorball CW, Jarvinen A. Elevated soluble urokinase plasminogen activator receptor (suPAR) predicts mortality in Staphylococcus aureus bacteremia. European journal of clinical microbiology & infectious diseases : official publication of the European Society of Clinical Microbiology 2011;30:1417-24.

15. Persson M, Ostling G, Smith G, et al. Soluble urokinase plasminogen activator receptor: a risk factor for carotid plaque, stroke, and coronary artery disease. Stroke; a journal of cerebral circulation 2014;45:18-23.

16. Raggam RB, Wagner J, Pruller F, et al. Soluble urokinase plasminogen activator receptor predicts mortality in patients with systemic inflammatory response syndrome. Journal of internal medicine 2014;276:651-8.

17. Reichsoellner M, Raggam RB, Wagner J, Krause R, Hoenigl M. Clinical evaluation of multiple inflammation biomarkers for diagnosis and prognosis for patients with systemic inflammatory response syndrome. Journal of clinical microbiology 2014;52:4063-6.

18. Suberviola B, Castellanos-Ortega A, Ruiz Ruiz A, Lopez-Hoyos M, Santibanez M. Hospital mortality prognostication In sepsis using the new biomarkers suPAR and proADM in a single determination on ICU admission. Intensive care medicine 2013;39:1945-52.

19. Zimmermann HW, Koch A, Seidler S, Trautwein C, Tacke F. Circulating soluble urokinase plasminogen activator is elevated in patients with chronic liver disease, discriminates stage and aetiology of cirrhosis and predicts prognosis. Liver international : official journal of the International Association for the Study of the Liver 2012;32:500-9.

20. Donadello K, Scolletta S, Taccone FS, et al. Soluble urokinase-type plasminogen activator receptor as a prognostic biomarker in critically ill patients. Journal of critical care 2014;29:144-9.

21. Haupt TH, Petersen J, Ellekilde G, et al. Plasma suPAR levels are associated with mortality, admission time, and Charlson Comorbidity Index in the acutely admitted medical patient: a prospective observational study. Critical care (London, England) 2012;16:R130.

22. Eugen-Olsen J, Andersen O, Linneberg A, et al. Circulating soluble urokinase plasminogen activator receptor predicts cancer, cardiovascular disease, diabetes and mortality in the general population. Journal of internal medicine 2010;268:296-308.

23. Lyngbaek S, Marott JL, Sehestedt T, et al. Cardiovascular risk prediction in the general population with use of suPAR, CRP, and Framingham Risk Score. International journal of cardiology 2013;167:2904-11.

24. Langkilde A, Hansen TW, Ladelund S, et al. Increased plasma soluble uPAR level is a risk marker of respiratory cancer in initially cancer-free individuals. Cancer epidemiology, biomarkers & prevention : a publication of the American Association for Cancer Research, cosponsored by the American Society of Preventive Oncology 2011;20:609-18.

25. Chounta A, Ellinas C, Tzanetakou V, et al. Serum soluble urokinase plasminogen activator receptor as a screening test for the early diagnosis of hepatocellular carcinoma. Liver international : official journal of the International Association for the Study of the Liver 2015;35:601-7.

**Table 1**

| Hospital | Period 1 | Period 2 | Period 3 | Period 4 | Period 5 | Period 6 |
| --- | --- | --- | --- | --- | --- | --- |
| Herlev | + suPAR | Control | Control | + suPAR | Control | Control |
| Bispebjerg | Control | + suPAR | Control | Control | + suPAR | Control |
| Hillerød | Control | Control | + suPAR | Control | Control | + suPAR |

**Figure 1:**

**suPAR level and risk of mortality in acutely admitted medical patients**

Patients < 70 years:

| **Mortality risk (% (95%CI)) stratified according to suPAR levels in patients below 70 years** | | |
| --- | --- | --- |
| **suPAR (ng/ml)** | **30 days** | **90 days** |
| **Alle (n=2652)** | **1.5% (1.1-2.0)** | **2.9% (2.2-3.6)** |
| 0-3 (n=1705) | **0.3 %** (0.1-0.7; n=5) | **0.8 %** (0.5-1.4; n=14) |
| 3-6 (n=744) | **1.6 %** (0.8-2.8; n=12) | **3.6 %** (2,4-5,2; n=27) |
| 6-9 (n=127) | **7.1 %** (3.3-13.0; n=9) | **11.8 %** (6.8-18.7; n=15) |
| >9 (n=76) | **19.7 %** (11.5-30.5; n=15) | **27.6 %** (18.0-39.1; n=21) |

**Examples:**

A man/women of 63 years with a suPAR level of 2.7 ng/ml has a risk of mortality within 30 days of 0.3%, which is five times less than the average mortality (1.5%) in patients below 70 years of age.

A man/women of 63 years with a suPAR level of 7.7 ng/ml has a risk of mortality within 30 days of 7.1%, which is five times the average mortality (1.5%) in patients below 70 years of age.

Patients > 70 years:

| **Mortality risk (% (95%CI)) stratified according to suPAR levels in patients above 70 years** | | |
| --- | --- | --- |
| **suPAR ng/ml** | **30 days** | **90 days** |
| **Alle (n=1691)** | **10.8% (9.3-12.3)** | **18,3% (16.4-20.1)** |
| 0-3 (n=339) | **2.7 %** (1.2-5.0; n=9) | **3,5 %** (1.8-6.1; n=12) |
| 3-6 (n=913) | **7.1 %** (5.5-9.0; n=65) | **13,7 %** (11.5-16.1; n=125) |
| 6-9 (n=293) | **19.8 %** (15.4-24.8; n=58) | **32,8 %** (27.4-38.5; n=96) |
| >9 (n=146) | **34.9 %** (27.2-43.3; n=51) | **52,1 %** (43.6-60.4; n=76) |

**Examples**:

A man/women of 75 years with a suPAR level of 2.7 ng/ml has a risk of mortality within 30 days of 2.7%, which is five times less than the average mortality (10.8%) in patients above 70 years of age.

A man/women of 75 years with a suPAR level of 7.7 ng/ml has a risk of mortality within 30 days of 19.8%, which is twice as high as the average mortality (10.8%) in patients above 70 years of age.

Source: Data is calculated from a cohort of acutely admitted medical patients (n=4343) from the emergency department at Hvidovre Hospital, from November 18 2013 until March 31 2014.

# Final Protocol (in Danish)

**Introduktion af suPAR-måling i akutmodtagelser**

**(TRIAGE III)**

Teknisk protokol V.5 (Final)

13. Mar. 2016 /MSC

**Generelle oplysninger**

**Styregruppe**

Seniorforsker, phd Jesper Eugen-Olsen, Hvidovre Hospital.

Professor, dr.med Lars S Rasmussen, Rigshospitalet.

Professor, dr.med Lars Køber, Rigshospitalet.

Overlæge, dr.med Kasper Iversen, Herlev Hospital

Overlæge dr.med Erik Kjøller, Herlev Hospital

Ledende overlæge Lisbet Ravn, Herlev Hospital

Ledende overlæge Birgitte Nybo Jensen, Bispebjerg Hospital

**Lokalisation**

Projektet udføres på Herlev Hospital og Bispebjerg Hospital

**Tidsplan**

Planlagt projektstart er 11. januar 2016 og ventes afsluttet 6. juni 2016 kl 08.00

**Baggrund**

Risikovurdering er central ved modtagelse og vurderingen af akut indlagte patienter skal sikre, at de mest akut syge bliver behandlet først og observeres tættest. Risikovurdering foretages i dag ved hjælp af systematiseret triage. Systematiseret triage foregår typisk ved en gennemgang af patientens primære symptom samt deres vitale parametre. Både symptom og vitale parametre tillægges en numerisk værdi ud fra et scoringssystem, bestemt af triagemodellen.  På denne måde opdeles patienterne i kategorier, som vha. farver eller tal signalerer, hvor hurtigt de skal tilses, samt hvor tæt de skal observeres^1^.

Gennem de seneste år er der kommet tiltagende fokus på, om man kan forbedre risikovurderingen i hospitalernes modtagelser ved at addere resultater fra biomarkørmålinger til den eksisterende triage. Det ser ud som om, at en sådan kombineret strategi er brugbar både ved specifikke diagnoser^2-4^ og mere generelt^5-7^. Hvilke biomarkører der skal bruges, og om det rent faktisk gør en forskel for patienterne, er der indtil videre ingen data på.

Soluble urokinase plasminogen activating receptor (suPAR) er den opløselige form af urokinase-type plasminogen activator receptor (uPAR) og primært udtrykt på immunceller som neutrofile leukocytter, aktiverede T-celler og makrofager^8^. Studier af suPAR har vist, at proteinet er stærkt associeret med mortalitet og morbiditet i en lang række specifikke sygdomme (sepsis, leversygdom, hjertesygdom, lungesygdom)^9-19^, generelt ved indlagte patienter^20,21^ og i den generelle befolkning^22,23^. Derudover har suPAR vist sig at kunne identificere patienter med manifest eller med høj risiko for at udvikle cancer^24,25^

Det ser således ud til, at suPAR er helt uspecifik og derfor ikke kan bruges til at diagnosticere nogen bestemt sygdom, men udelukkende til at identificere lav- og høj-risikopatienter generelt. Dermed kunne suPAR potentielt være en god markør i akutmodtagelser til at identificere risikopatienter, der kræver tættere observation, hurtigere behandling og/eller videre udredning. På den anden side ser det også ud til at patienter med lav suPAR har en overordentlig god prognose, og suPAR vil derfor potentielt også kunne identificere patienter, der hurtigt kan udskrives. Enkelte steder (eksempelvis Hvidovre Hospital) har man allerede indført suPAR som en rutinemarkør, der tages på alle indlagte patienter i akutmodtagelsen.

Selvom det er vist, at suPAR både kan identificere høj- og lavrisiko-patienter, er det aldrig vist, at man kan bruge denne viden til at forebygge dødsfald eller alvorlige komplikationer og ej heller om det kan forbedre patient flowet.

**Formål**

At undersøge om måling af suPAR og information herom til læger, der arbejder i akutmodtagelsen, kan forebygge dødsfald eller alvorlige komplikationer samt forbedre patient flow hos akut indlagte medicinske og kirurgiske patienter i akutmodtagelsen.

**Metode**

Studiet er et åbent overkrydsnings cluster-randomiseret interventionsstudie af betydningen af suPAR-måling i akutmodtagelsen.

**Målepunkter**

*Primære endepunkt:*

- Død af alle årsager opgjort 10 måneder efter inklusion af sidste patient.

*Sekundære endepunkter:*

- Død inden for en måned
- Antal patienter udskrevet indenfor 24 timer
- Indlæggelser på medicinske afdelinger indenfor 30 dage
- Indlæggelser på intensivafdeling indenfor 30 dage
- Nye cancerdiagnoser opgjort 10 måneder efter inklusion af sidste patient
- Indlæggelsesvarighed
- Akutte genindlæggelser inden for 30 og 90 dage
- Justerede analyser for aldersgrupper og specifikke sygdomme med hensyn til center, A-diagnose og køn.

**Patienter:**

suPAR indføres som rutinemarkør på alle patienter, der indlægges akut i akutmodtagelsen på Bispebjerg- og Herlev Hospital og får taget blodprøver i 3 x 21 dages perioder på de 2 matrikler. Perioder på 21 dage før, imellem og efter studieperioderne fungerer som kontrolperioder (Tabel 1).

*Inklusionskriterier:* Patienter der indlægges akut og som får taget Hæmoglobin, CRP og Kreatinin indenfor 6 timer efter registrering i det grønne system. Alder på 16 eller over.

*Eksklusionskriterier:* Patienter som indlægges i gyn./obs.- og pædiatri regi eksluderes.

**Måling af suPAR**

Materiale til måling af suPAR tages fra i forbindelse med rutine-blodprøvetagning og medfører ikke ekstra stik til patienter.

suPAR måles på EDTA-plasma fra overskydende materiale fra rutineblodprøverne. Analysen udføres med suPARnostic^®^ Quick Triage stick (QT stick). Der udtages 10 mikroliter (µl) EDTA-plasma fra rutine-blodprøverne. Disse 10 µl overføres til et præfabrikeret rør, der indeholder 100 µl ”running buffer”. Herfra pipetteres 60 µl på QT sticken, som sættes i analyseapparatet, og der trykkes start. Efter 20 minutter aflæses resultatet automatisk og rapporteres på den tilkoblede PC. Den benyttede QT stick smides ud, og apparatet er klar til ny måling. Der opstilles 3 analyseapparater, således at 3 analyser kan køres sideløbende, og der kan som minimum analyseres 9 prøver i timen. Så snart resultatet på suPAR foreligger på den til analyseapparatet tilkoblede PC, vil svaret blive skrevet ind i de elektroniske blodprøve- og journalsystemer LABKA og OPUS. Det tilstræbes, at alle suPAR-analysesvar er indskrevet i LABKA/OPUS hurtigst muligt og max 2 timer efter blodprøvetagning.

Afpippetering og analyse af suPAR foretages af studiepersonale der er til stede døgnet rundt.

**Information til hospitalets læger om suPAR**

Forud for studieperioden vil læger på de implicerede hospitaler blive informeret skriftligt om den prognostiske betydning af suPAR både i forhold til specifikke diagnoser og i forhold til uselekterede patienter. Der vil desuden blive tilbudt undervisning på de enkelte afdelinger på sygehuset. Alle læger vil få tilsendt et resume over de data, der forligger om suPAR’s prognostiske betydning, og få udleveret lommekort, der beskriver risiko for mortalitet ved 30- og 90-dage ved forskellige niveauer af suPAR, baseret på data fra Hvidovre - og Nordsjælland Hospital.

**Dataopsamling**

Resultater fra suPAR-målingen samt øvrige biokemiske målinger vil være registreret i LABKA-databasen, og data vil blive trukket herfra. Information om primært og sekundære endepunkter indhentes fra centrale registre.

**Styrkeberegning**

I et tidligere endnu upubliceret studie af patienter indlagt i akutmodtagelsen på Nordsjælland Hospital var dødeligheden inden for 12 måneder 12,7 %. Vores hypotese er, at vi kan reducere mortaliteten til 11,2 % ved at måle suPAR på indlagte patienter. På samme kohorte var Genindlæggelsesprocenten 16 % efter 5 måneder. Ved et signifikansniveau på 5 % og en power på 80% skal man, såfremt grupperne er lige store, bruge en stikprøvestørrelse på 7340 personer i hver gruppe for at vise denne forskel.

Baseret på tal fra de 2 akutmodtagelser indlægges der ca. 170 patienter dagligt på de 2 hospitaler tilsammen. I studieperioden vil der således kunne opsamles data på 10.700 patienter i både den aktive og i kontrolarmen. Dette vil være tilstrækkeligt til at undersøge den primære hypotese.

**Tilladelser**

Studiet er godkendt af direktionerne på de deltagende hospitaler samt af de enkelte afdelingsledelser. Studiet er vurderet, men ikke fundet anmeldelsespligtigt af videnskabsetisk komité i henhold til gældende lovgivning (j. nummer FSP-15003590). Databehandling er anmeldt til og godkendt af Datatilsynet( HGH-2015-042 I-Suite nr. 04087) og vil blive anmeldt til Sundhedsstyrelsen. Studiet er registeret på [clinicaltrials.gov](http://clinicaltrials.gov) (NCT02643459).

**Økonomi**

Udgifter i forbindelse med studiet er finansieret af firmaet ViroGates A/S. Der vil sammen med ViroGates A/S blive søgt ekstern finansiering til dele af eller hele projektet.

**Referencer**

1. Farrohknia N, Castren M, Ehrenberg A, et al. Emergency department triage scales and their components: a systematic review of the scientific evidence. Scandinavian journal of trauma, resuscitation and emergency medicine 2011;19:42.

2. Albrich WC, Ruegger K, Dusemund F, et al. Biomarker-enhanced triage in respiratory infections: a proof-of-concept feasibility trial. The European respiratory journal 2013;42:1064-75.

3. Sibon I, Rouanet F, Meissner W, Orgogozo JM. Use of the Triage Stroke Panel in a neurologic emergency service. The American journal of emergency medicine 2009;27:558-62.

4. Vanni S, Polidori G, Pepe G, et al. Use of biomarkers in triage of patients with suspected stroke. The Journal of emergency medicine 2011;40:499-505.

5. Gans SL, Atema JJ, Stoker J, Toorenvliet BR, Laurell H, Boermeester MA. C-reactive protein and white blood cell count as triage test between urgent and nonurgent conditions in 2961 patients with acute abdominal pain. Medicine 2015;94:e569.

6. Herlitz J, Svensson L. The value of biochemical markers for risk stratification prior to hospital admission in acute chest pain. Acute cardiac care 2008;10:197-204.

7. Seymour CW, Cooke CR, Wang Z, et al. Improving risk classification of critical illness with biomarkers: a simulation study. Journal of critical care 2013;28:541-8.

8. Huai Q, Mazar AP, Kuo A, et al. Structure of human urokinase plasminogen activator in complex with its receptor. Science (New York, NY) 2006;311:656-9.

9. Borne Y, Persson M, Melander O, Smith JG, Engstrom G. Increased plasma level of soluble urokinase plasminogen activator receptor is associated with incidence of heart failure but not atrial fibrillation. European journal of heart failure 2014;16:377-83.

10. Eapen DJ, Manocha P, Ghasemzedah N, et al. Soluble urokinase plasminogen activator receptor level is an independent predictor of the presence and severity of coronary artery disease and of future adverse events. Journal of the American Heart Association 2014;3:e001118.

11. Gumus A, Altintas N, Cinarka H, et al. Soluble urokinase-type plasminogen activator receptor is a novel biomarker predicting acute exacerbation in COPD. International journal of chronic obstructive pulmonary disease 2015;10:357-65.

12. Lyngbaek S, Andersson C, Marott JL, et al. Soluble urokinase plasminogen activator receptor for risk prediction in patients admitted with acute chest pain. Clinical chemistry 2013;59:1621-9.

13. Lyngbaek S, Marott JL, Moller DV, et al. Usefulness of soluble urokinase plasminogen activator receptor to predict repeat myocardial infarction and mortality in patients with ST-segment elevation myocardial infarction undergoing primary percutaneous intervention. The American journal of cardiology 2012;110:1756-63.

14. Molkanen T, Ruotsalainen E, Thorball CW, Jarvinen A. Elevated soluble urokinase plasminogen activator receptor (suPAR) predicts mortality in Staphylococcus aureus bacteremia. European journal of clinical microbiology & infectious diseases : official publication of the European Society of Clinical Microbiology 2011;30:1417-24.

15. Persson M, Ostling G, Smith G, et al. Soluble urokinase plasminogen activator receptor: a risk factor for carotid plaque, stroke, and coronary artery disease. Stroke; a journal of cerebral circulation 2014;45:18-23.

16. Raggam RB, Wagner J, Pruller F, et al. Soluble urokinase plasminogen activator receptor predicts mortality in patients with systemic inflammatory response syndrome. Journal of internal medicine 2014;276:651-8.

17. Reichsoellner M, Raggam RB, Wagner J, Krause R, Hoenigl M. Clinical evaluation of multiple inflammation biomarkers for diagnosis and prognosis for patients with systemic inflammatory response syndrome. Journal of clinical microbiology 2014;52:4063-6.

18. Suberviola B, Castellanos-Ortega A, Ruiz Ruiz A, Lopez-Hoyos M, Santibanez M. Hospital mortality prognostication in sepsis using the new biomarkers suPAR and proADM in a single determination on ICU admission. Intensive care medicine 2013;39:1945-52.

19. Zimmermann HW, Koch A, Seidler S, Trautwein C, Tacke F. Circulating soluble urokinase plasminogen activator is elevated in patients with chronic liver disease, discriminates stage and aetiology of cirrhosis and predicts prognosis. Liver international : official journal of the International Association for the Study of the Liver 2012;32:500-9.

20. Donadello K, Scolletta S, Taccone FS, et al. Soluble urokinase-type plasminogen activator receptor as a prognostic biomarker in critically ill patients. Journal of critical care 2014;29:144-9.

21. Haupt TH, Petersen J, Ellekilde G, et al. Plasma suPAR levels are associated with mortality, admission time, and Charlson Comorbidity Index in the acutely admitted medical patient: a prospective observational study. Critical care (London, England) 2012;16:R130.

22. Eugen-Olsen J, Andersen O, Linneberg A, et al. Circulating soluble urokinase plasminogen activator receptor predicts cancer, cardiovascular disease, diabetes and mortality in the general population. Journal of internal medicine 2010;268:296-308.

23. Lyngbaek S, Marott JL, Sehestedt T, et al. Cardiovascular risk prediction in the general population with use of suPAR, CRP, and Framingham Risk Score. International journal of cardiology 2013;167:2904-11.

24. Langkilde A, Hansen TW, Ladelund S, et al. Increased plasma soluble uPAR level is a risk marker of respiratory cancer in initially cancer-free individuals. Cancer epidemiology, biomarkers & prevention : a publication of the American Association for Cancer Research, cosponsored by the American Society of Preventive Oncology 2011;20:609-18.

25. Chounta A, Ellinas C, Tzanetakou V, et al. Serum soluble urokinase plasminogen activator receptor as a screening test for the early diagnosis of hepatocellular carcinoma. Liver international : official journal of the International Association for the Study of the Liver 2015;35:601-7.

**Figur 1**


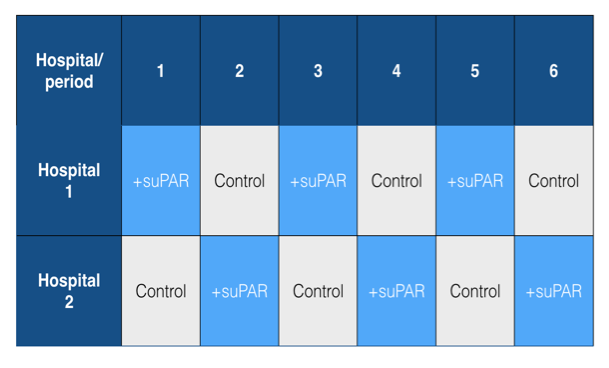


# Final Protocol (translated from Danish)

**Introducing suPAR measurement in the**

**emergency departments**

**(TRIAGE III)**

Technical protocol V.5 (Final)

March 13 2016 /MSC

**General information**

**Steering committee:**

Senior researcher, PhD Jesper Eugen-Olsen, Hvidovre Hospital

Professor, DMSc. Lars S Rasmussen, Rigshospitalet

Professor, DMSc. Lars Køber, Rigshospitalet

M.D., DMSc. Kasper Iversen, Herlev Hospital

M.D., DMSc. Erik Kjøller, Herlev Hospital

Chief Physician Lisbet Ravn, Herlev Hospital

Chief Physician Birgitte Nybo Jensen, Bispebjerg Hospital

**Location**

The study will take place at Herlev Hospital and Bispebjerg Hospital.

**Time plan**

Planned start of inclusion is on January 11 2016 and is expected to be concluded on June 6 2016.

**Background**

Risk assessment is a central concept at admittance of acutely admitted patients to ensure that the sickest patients are treated first and observed the closest. Today, systematized risk assessment is being performed with triage algorithms. According to the primary complaint and vitals sign of the patient an aggregated score is determined by the triage algorithm. Patients are then stratified in groups of acuity that signals how fast they should be assessed by doctors and the frequency of observation^1^. In recent years it has been suggested that the risk assessment in the emergency departments can be strengthened by adding results from blood-based biomarkers to the triage process. A combined strategy looks viable both in specific diagnoses^2-4^ and in general^5-7^. However, there is no data to support whether this strategy makes a difference to the patients or which biomarkers that should be used.

Soluble urokinase plasminogen activating receptor (suPAR) is the soluble form of the urokinase-type plasminogen activator receptor (uPAR), which primary is expressed on the surface of cells in the immune system (Neutrophiles cells, activated T-cells and macrophages)^8^. Studies have shown that suPAR is strongly associated with mortality and morbidity in a wide range of specific diseases (sepsis, hepatic-, cardiac- and lung-diseases) ^9-19^, among admitted patients^20,21^ and in the general population^22,23^. In addition suPAR have been shown to identify patients at low and high risk of developing cancer^24,25^. It appears that suPAR is a nonspecific marker of outcome and not suited as a diagnostic tool but solely to identify patients at low and high risk.

Thus, suPAR could potentially be a good marker in the emergency department to identify risk-patients requiring close observation, fast treatment and further investigation. On the other hand, it appears that patients with low suPAR level have a very good prognosis and are potentially suitable for rapid discharge. In few places (e.g. Hvidovre Hospital) suPAR have already been implemented as a routine analysis of all acutely admitted medical patients.

Although it has been shown that suPAR is suitable for identification of patients at high and low risk, it has never been shown if this information can be used to prevent deaths or serious complications, nor if it can improve patient flow in the emergency department (ED).

**Objective**

To assess if measurement of the suPAR level and information to the attending ED doctors can prevent serious complications and deaths among acutely admitted medical- and surgical patients and improve flow in the emergency department.

**Methods**

Triage III is an open, cross-over, cluster-randomized, interventional study on the impact of introducing suPAR measurement in the emergency department.

**Outcomes**

*Primary outcome:*

- All-cause mortality assessed 10 months prior to inclusion of the last patient

*Secondary outcomes:*

- All-cause mortality at one month follow-up
- Number of patients discharged within 24 hours
- Number of admissions at the medical wards within 30 days
- Number of transfers to the intensive care unit within 30 days
- New cancer diagnoses assessed 10 months prior to inclusion of the last patient
- Length of stay
- Number of readmissions within 30 and 90 days
- Analyses adjusted for gender, age, hospital and specific diseases.

**Patients:**

suPAR will be introduced as a routine analysis on all acutely admitted patients at the emergency department at Herlev – and Bispebjerg hospital in three 21-day periods on both hospitals. Periods of 21 days before, in between, and after will serve as control periods (Figure 1).

*Inclusion criteria:* Acutely admitted patients, who have routine blood-tests (including hemoglobin, C-reactive protein and creatinine) analyzed within six hours of registration. Age of 16 years or above.

*Exclusion criteria:* Patients admitted in gynecological-/obstetrics-/pediatric regimen.

**Measurement of suPAR**

Plasma for suPAR measurement will be obtained in connection with routine blood test and will not result in additional discomfort for the patients.

The suPAR level is measured on excess EDTA-plasma from the routine blood tests. The analysis is done with the suPARnostic^®^ Quick Triage stick (QT stick). From routine blood-tests 10 microliter (µl) EDTA-plasma is extracted and transferred to a prefabricated test tube containing 100 µl ”running buffer”. From the mixture, 60 µl is pipetted to the QT stick which is inserted in the analyzer. The suPAR level is automatically reported on the connected PC after 20 minutes. During the inclusion period it is possible to analyze three tests simultaneously at a minimum of nine tests an hour. When the test result is available it will be reported to the electronic Laboratory system ”LABKA” and electronical hospital records (“OPUS”) as soon as possible and within two hours. Measurement of suPAR is done by study staff, which is present 24 hours every day during inclusion.

**Information to attending doctors regarding the suPAR level**

Prior to the study period, doctors will receive written information regarding the prognostic value of suPAR in general and with regard to specific diagnoses. In addition, all participating departments will be offered formalized teaching sessions. Doctors will receive a review of the published literature and pocket cards describing risk of 30- and 90-day mortality depending on the suPAR level based on data from Hvidovre Hospital and Hillerød hospital

**Data**

SuPAR levels as well as all other biochemical test are registered in the LABKA database and can be extracted. Data on all outcomes will be obtained from the central Danish registries.

**Power calculation**

In a previous unpublished study of patients acutely admitted to the ED at Copenhagen University Hospital North Zealand, Denmark, 12-month mortality was 12.7 % and the frequency of readmissions was 16 % within 5 months.

Our main hypothesis is that we can reduce all-cause mortality to 11.2% by measuring suPAR on admitted patients. Using a 5 % level of significance and a power of 80 %, we will need a sample of 7340 patients in each randomization group to detect this difference an absolute risk reduction in mortality at least 10 months after admission of 1.5 %.

Based upon numbers from the two emergency departments, approximately 170 patients are admitted on a daily basis. During the inclusion period it will be possible to collect data on 10,700 patients in both active- and control periods. This will be sufficient to assess the primary hypothesis.

**Ethics and approvals**

The study was approved by the board of directors at each participating hospital and department heads of the emergency departments gave consent. The study was presented to the Regional Ethics Committee, who decided that no formal approval was needed in accordance with Danish law (Jr. Nr.: FSP-15003590). All processing of personal data followed national guidelines in accordance with Danish law and the study was approved by the Danish Data Protection Agency (HGH-2015-042, I-Suite nr.04087) and will be registered at the Danish Patient Safety Authority. The study is registered at clinicaltrials.org (NCT02643459).

**Economy**

Expenses related to the study are financed by ViroGates A / S. Together with ViroGates external funding will be sought for parts of or the entire project.

**References**

1. Farrohknia N, Castren M, Ehrenberg A, et al. Emergency department triage scales and their components: a systematic review of the scientific evidence. Scandinavian journal of trauma, resuscitation and emergency medicine 2011;19:42.

2. Albrich WC, Ruegger K, Dusemund F, et al. Biomarker-enhanced triage in respiratory infections: a proof-of-concept feasibility trial. The European respiratory journal 2013;42:1064-75.

3. Sibon I, Rouanet F, Meissner W, Orgogozo JM. Use of the Triage Stroke Panel in a neurologic emergency service. The American journal of emergency medicine 2009;27:558-62.

4. Vanni S, Polidori G, Pepe G, et al. Use of biomarkers in triage of patients with suspected stroke. The Journal of emergency medicine 2011;40:499-505.

5. Gans SL, Atema JJ, Stoker J, Toorenvliet BR, Laurell H, Boermeester MA. C-reactive protein and white blood cell count as triage test between urgent and nonurgent conditions in 2961 patients with acute abdominal pain. Medicine 2015;94:e569.

6. Herlitz J, Svensson L. The value of biochemical markers for risk stratification prior to hospital admission in acute chest pain. Acute cardiac care 2008;10:197-204.

7. Seymour CW, Cooke CR, Wang Z, et al. Improving risk classification of critical illness with biomarkers: a simulation study. Journal of critical care 2013;28:541-8.

8. Huai Q, Mazar AP, Kuo A, et al. Structure of human urokinase plasminogen activator in complex with its receptor. Science (New York, NY) 2006;311:656-9.

9. Borne Y, Persson M, Melander O, Smith JG, Engstrom G. Increased plasma level of soluble urokinase plasminogen activator receptor is associated with incidence of heart failure but not atrial fibrillation. European journal of heart failure 2014;16:377-83.

10. Eapen DJ, Manocha P, Ghasemzedah N, et al. Soluble urokinase plasminogen activator receptor level is an independent predictor of the presence and severity of coronary artery disease and of future adverse events. Journal of the American Heart Association 2014;3:e001118.

11. Gumus A, Altintas N, Cinarka H, et al. Soluble urokinase-type plasminogen activator receptor is a novel biomarker predicting acute exacerbation in COPD. International journal of chronic obstructive pulmonary disease 2015;10:357-65.

12. Lyngbaek S, Andersson C, Marott JL, et al. Soluble urokinase plasminogen activator receptor for risk prediction in patients admitted with acute chest pain. Clinical chemistry 2013;59:1621-9.

13. Lyngbaek S, Marott JL, Moller DV, et al. Usefulness of soluble urokinase plasminogen activator receptor to predict repeat myocardial infarction and mortality in patients with ST-segment elevation myocardial infarction undergoing primary percutaneous intervention. The American journal of cardiology 2012;110:1756-63.

14. Molkanen T, Ruotsalainen E, Thorball CW, Jarvinen A. Elevated soluble urokinase plasminogen activator receptor (suPAR) predicts mortality in Staphylococcus aureus bacteremia. European journal of clinical microbiology & infectious diseases : official publication of the European Society of Clinical Microbiology 2011;30:1417-24.

15. Persson M, Ostling G, Smith G, et al. Soluble urokinase plasminogen activator receptor: a risk factor for carotid plaque, stroke, and coronary artery disease. Stroke; a journal of cerebral circulation 2014;45:18-23.

16. Raggam RB, Wagner J, Pruller F, et al. Soluble urokinase plasminogen activator receptor predicts mortality in patients with systemic inflammatory response syndrome. Journal of internal medicine 2014;276:651-8.

17. Reichsoellner M, Raggam RB, Wagner J, Krause R, Hoenigl M. Clinical evaluation of multiple inflammation biomarkers for diagnosis and prognosis for patients with systemic inflammatory response syndrome. Journal of clinical microbiology 2014;52:4063-6.

18. Suberviola B, Castellanos-Ortega A, Ruiz Ruiz A, Lopez-Hoyos M, Santibanez M. Hospital mortality prognostication in sepsis using the new biomarkers suPAR and proADM in a single determination on ICU admission. Intensive care medicine 2013;39:1945-52.

19. Zimmermann HW, Koch A, Seidler S, Trautwein C, Tacke F. Circulating soluble urokinase plasminogen activator is elevated in patients with chronic liver disease, discriminates stage and aetiology of cirrhosis and predicts prognosis. Liver international : official journal of the International Association for the Study of the Liver 2012;32:500-9.

20. Donadello K, Scolletta S, Taccone FS, et al. Soluble urokinase-type plasminogen activator receptor as a prognostic biomarker in critically ill patients. Journal of critical care 2014;29:144-9.

21. Haupt TH, Petersen J, Ellekilde G, et al. Plasma suPAR levels are associated with mortality, admission time, and Charlson Comorbidity Index in the acutely admitted medical patient: a prospective observational study. Critical care (London, England) 2012;16:R130.

22. Eugen-Olsen J, Andersen O, Linneberg A, et al. Circulating soluble urokinase plasminogen activator receptor predicts cancer, cardiovascular disease, diabetes and mortality in the general population. Journal of internal medicine 2010;268:296-308.

23. Lyngbaek S, Marott JL, Sehestedt T, et al. Cardiovascular risk prediction in the general population with use of suPAR, CRP, and Framingham Risk Score. International journal of cardiology 2013;167:2904-11.

24. Langkilde A, Hansen TW, Ladelund S, et al. Increased plasma soluble uPAR level is a risk marker of respiratory cancer in initially cancer-free individuals. Cancer epidemiology, biomarkers & prevention : a publication of the American Association for Cancer Research, cosponsored by the American Society of Preventive Oncology 2011;20:609-18.

25. Chounta A, Ellinas C, Tzanetakou V, et al. Serum soluble urokinase plasminogen activator receptor as a screening test for the early diagnosis of hepatocellular carcinoma. Liver international : official journal of the International Association for the Study of the Liver 2015;35:601-7.

**Figure 1:**


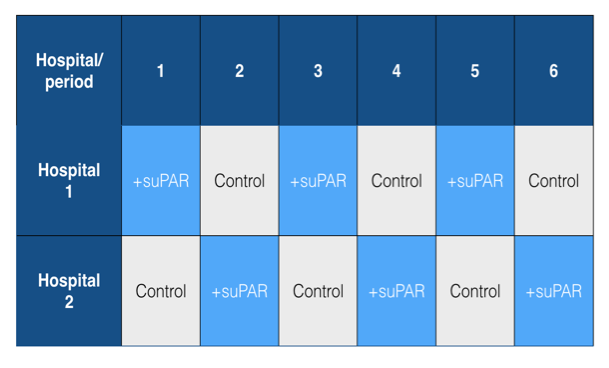


# Original Statistical analysis plan

*The original statistical analysis plan was published with the design article on August 5 2016.*

Patients admitted in each intervention or control cycle will be followed as a single cohort and data will be analyzed as randomized. The two groups will be assessed for comparability of the following variables: age, sex, and Charlson score. Differences in mean age of more than 5 years and/or an absolute Charlson Comorbidity Index score of 2 or more will be adjusted for in the final analysis.

Patient data will be analyzed according to which arm of the trial the patient is admitted to during index admission according to the randomization scheme and corresponding to the intention-to-treat principle.

A weighted Cox model will be used to compare mortality at 10 months after inclusion of the last patient. Patients are censored if their first readmission is in the opposite group of their index admission. As this censoring is likely to be dependent censoring (a readmission is rarely a positive prognostic signal), we will employ Inverse Probability of Censoring Weighting (IPCW) where patients readmitted to their own treatment group will be up-weighted to compensate. We will employ stabilized weights such that the reweighted sample has the same implied sample size throughout follow-up. Due to the design, time since index admission is the only covariate that needs to be included in the weights. Reweighing will be done for every two weeks of follow-up. We will not censor nor reweight for 2nd or later readmissions, since the weights would become highly unstable and it is not likely that the presence or absence of an initial suPAR measurement will be important for clinical decisions at this stage.

Furthermore, a traditional intention-to-treat analysis will be performed. Notable difference between the results of the two analysis strategies will be considered critically.

Kaplan-Meier plots will be used to illustrate survival. Unpaired T-test will be used to compare length of stay. P < 0.05 will be considered significant.

Subgroup analysis of the following groups will be performed: patients aged 65 year and above, and patients discharged with diagnoses of surgical conditions, cancer, infections, and cardiovascular disease.

# Final Statistical analysis plan

**April 5 2017**

This document contains the statistical analysis plan for the TRIAGE III trial. The statistical analysis plan has been completed prior to the availability of the outcome data. The plan was made available on April 5 2017 on a public website:

<https://www.hvidovrehospital.dk/afdelinger-og-klinikker/Klinisk-Forskningscenter/om-centret/Til-forskere-paa-hospitalet/Documents/TRIAGE%20III%20Analysis%20plan.pdf>

and at [clinicaltrials.gov](http://clinicaltrials.gov).

**Time-line:**

Study start: January 11 2016. Study completion: June 6 2016. **The primary analysis will be conducted 10 months after inclusion of the last patient**, which is expected to be April 6 2017.

**Background:**

The primary research question is: **Can the introduction of a prognostic biomarker strengthen risk stratification of acutely admitted patients leading to fewer deaths.**

The study is a cluster randomized controlled interventional trial conducted at two major emergency departments of unselected patients acutely admitted.

**Design:**

Cluster-randomized, cross-over, parallel interventional two center trial, conducted around six cycles of three weeks in a 1:1 allocation ratio of intervention versus control at each cluster.

**Inclusion:**

1. ≥16 years at admittance.
2. Acute arrival at the emergency department within the inclusion period.
3. Have routine blood tests analyzed, including hemoglobin, C-reactive protein, and creatinine, within 6 hours of admission to the emergency department.
4. As the results of the blood tests can take up to two hours to provide, the doctors cannot change outcome based on biomarker levels until those data are provided. Hence, inclusion time will be calculated as time for blood sampling + 2 hours.

**Exclusion:**

1. Arrival at the gynecological-, obstetric-, and pediatric department.

**Ethics:**

The study was presented to the Regional Ethics Committee, who decided that no formal approval was needed in accordance with Danish law. The study has been approved by the Danish Patient Safety Authority (3-3013-1744/1) and data management has been approved by the Danish Data Protection Agency (ID: HGH-2015-042, I-suite: 04087).

**Study variables and endpoints:**

The following data are collected for the included patients:

- Age
- Results of the routine blood tests at the index admission including suPAR where available. Routine biomarkers: Hemoglobin, Leukocytes, thrombocytes, sodium, potassium, C-reactive protein, Creatinine, ALAT, Albumin, Calcium, lactate. suPAR in intervention periods
- Gender
- Personal Identification number (Danish Civil Registration number (CPR)
- At follow-up (10 months after inclusion of last patient) the following data is collected from the National Patient Registry (NPR)
- Contacts with the hospital system (including all historical contacts)
- Information regarding admissions (date, time and place of admission and discharge)
- Diagnoses (follow-up during study period, historical and in relation to index admission)
- Date of death or immigration (only very few immigrations are expected)
- Charlson score will be calculated using registered diagnoses in NPR dating two years back from index admission

**Sample size calculation**

In a previous unpublished study of patients acutely admitted to an Emergency Department in Denmark 12-month mortality was 12.7% and readmission rate was 16% within 5 months. Using a 5% level of significance and a power of 80%, the study will need to include 7,340 patients in each group to detect an absolute risk reduction in mortality of 1.5% at 10 months after admission. The two participating hospitals approximately admit 170 patients on a daily basis. During the study period approximately 10,700 patients will be included in every group.

**Study end**

The follow-up for all patients ends at April 6 2017 and this date will be used as the censoring time for the main analysis.

**Statistical plan for main outcome paper**

Statistical analyses will be performed using SAS and R.

**Consort diagram:**

A modified CONSORT trail profile will be used to describe patient flow and total number patients in each group. All patients not included in analysis will be explained.

**Baseline description of groups:**

Table of summary statistics for each group (described separately for each hospital in appendices) will be presented with a number of baseline variables (age, sex, results of routine blood tests, comorbidities).

Continuous variables will be summarized with: n (non-missing sample size), mean, standard deviation, median, interquartile range, number of missing values. Categorical variables will be reported as frequency and percentages (based on non-missing sample size) and number of missing values.

Continuous and ordinal variables will be tested by Wilcoxon analysis for differences between groups. Categorical variables will be tested with chi-square.

**Primary outcome analysis:**

The primary outcome will be an **analysis of all-cause mortality between the intervention and control groups**. Patients will be followed as one cohort and data will be analyzed as randomized. Patients will be included at their first contact with the emergency department (index admission) and are followed until the April 6 2017. Any patient lost to follow-up will be censored at the last time known to be alive.

Patients admitted in the intervention period without a suPAR measurement will remain in the intervention group for analyses corresponding to the intention-to-treat principle.

The main analysis of the primary outcome will use a weighted Cox model to compare the primary outcome of all-cause mortality. Patients are artificially censored if they are readmitted at one of the study emergency departments in the opposite group of their index admission. As this censoring is likely to be dependent censoring (a readmission is rarely a positive prognostic signal), we will employ Inverse Probability of Censoring Weighting (IPCW) where patients readmitted to their own treatment group will be up-weighted to compensate. We will employ stabilized weights such that the reweighted sample has the same implied sample size throughout follow-up. Due to the design, time since index admission is the only covariate that needs to be included in the weights. Reweighting will be done for every two weeks of follow-up. We will not censor nor reweight for 2nd or later readmissions, since the weights would become highly unstable and it is not likely that the presence or absence of an initial suPAR measurement will be important for clinical decisions at this stage.

A secondary analysis will be performed where the observational period ends if a patient is readmitted within the inclusion period. We anticipate, that the patients in the secondary analysis will seem healthier and the possible effect of the intervention weaker, although showing the same trend as the main analysis. Notable difference between the results of the main and the secondary analysis strategies will be considered critically.

Finally a per protocol weighted analysis is planned. In this analysis, only patients with a suPAR measurement are included in the intervention group. In order to investigate the difference between these results and the main analysis, we will analyze the mortality differences between patients with and without a suPAR measurement in the intervention periods.

Kaplan-Meier plots will be used to illustrate survival. Robust standard errors will be employed to account for the clustering. P < 0.05 will be considered statistically significant

**Adjusted analyses of primary endpoint:**

Three additional models will also be examined to further examine any role of differences in baseline values of the two groups. The first will include age, sex, CRP and center. The second will further include Charlson score. The third will include all variables used in the descriptive statistics. Results will be presented as hazard ratios with 95% confidence intervals. A forest plot will illustrate the results.

**Sensitivity analyses of the primary endpoint:**

Sensitivity analyses will be performed: One analysis where all readmissions are censored and a second analysis with only 10 months follow-up for all patients.

**Secondary endpoints:**

The predefined secondary endpoints of the study are: all-cause mortality at one month of admission; short admission (<24 h) to the emergency department; admission to the medical wards; admission to the intensive care unit; new (not previously registered) cancer diagnosis at the end of the study; length of stay during admission; readmission at one and three months; and economical expenses.

All-cause mortality at one month will be analyzed similar to the primary outcome. The discriminative ability of suPAR with regard to mortality at one and ten months will be assessed by using area under the curve for receiver operating characteristics curves as well as sensitivity, specificity, negative- and positive predictive value. To compare readmission risk, we will use Cox regression analysis. Cumulative incidence rates adjusted for competing risk (death, readmission) will be estimated. Unpaired t-test will be used to compare length of stay between groups.

**Subgroup analyses:**

Primary and secondary endpoints (mortality, readmissions, length of stay, discharge from the ED) will be analyzed in the following subgroups according to primary diagnosis at admission: cardiovascular disease, cancer, infections, neurological disease, surgery done during current admission, and patients aged 65 years or older. When Triage data is available, outcomes will be assessed according to DEPT triage categories, as suPAR previously has shown strongest prognostic effect in patients with lowest DEPT triage. All-cause mortality will be analyzed similar to the primary endpoint.

**Handling of missing data:**

The primary outcome analysis should be subject to no or little missing data as it is based on Danish register data.

**Handling of diagnoses for outcome and subgroups:**

Diagnoses obtained from the National Patient Registry are coded with the ICD-10 system. The original chapters will be used to group patients according to diagnoses. The primary diagnosis will be used when constructing subgroups and both primary and secondary diagnoses will be used to calculate Charlson score. The following will define the subgroups:

**Cancer:** Chapter II: Neoplasms (C00-C97 + D37-D48).

**Cardiovascular disease:** Chapter IX: Diseases of the circulatory system (I09-I52 + I70-I89)

**Infections:** Chapter I: **(**A00-B99 + J00-J22 + N10-N11+ N30-N31 + G00-G09)

**Neurological disease:** Chapter VI: Diseases of the nervous system (G09-G47 + I60-I69)

**Surgical conditions:** Presence of surgical procedure codes divided into different specialties (general, orthopedic, other)

# Summary of changes to the protocol

*Changes to the protocol were made before outcome data was available. The final protocol was published as a design article on August 5 2016:*

[*https://www.ncbi.nlm.nih.gov/pubmed/27491822*](https://www.ncbi.nlm.nih.gov/pubmed/27491822)

- The study was originally planned to be conducted at three separate hospitals. Due to internal factors and changes in management, Nordsjælland hospital could not continue to participate in the study and withdrew after four days of inclusion. Although attempts were made, it was not possible to find a replacement. Therefore the Steering Committee together with the chief statistician decided to change the study design to a two center model with 1:1 ratio of intervention and control. The new design had three active periods at each center instead of two, which meant that the inclusion period was prolonged until June 6 2016. A new power calculation reflected this change.
- The original follow-up period was planned to be 12 months, but based upon statistical advice the Steering Committee decided to adjust it to 10 months after inclusion of the last patient (median 12 months). It was also decided to include subgroups and adjusted analyses and to reduce the number of secondary endpoints.
- Inclusion- and exclusion-criteria were added.
- The reduction in centers also meant a reduced number of patients eligible for inclusion.

# Summary of changes to the Statistical Analysis Plan

*Changes to the Statistical Analysis Plan were made before outcome data was available.*

- Elaboration of the entire analysis plan.
- Added sensitivity and adjusted analyses of the primary outcome as well as a ”per-protocol” analysis.
- Specification of subgroup analyses and definitions of subgroups.

# Summary of changes at [clinicaltrials.gov](http://clinicaltrials.gov):

*Changes were made before outcome data was available.*

- February 5, 2016: Status of study changed to recruiting. The study started inclusion on January 11, 2016.
- March 1, 2016: The secondary endpoint “Economical expenses” was added after the Steering Committee decided on inclusion of this outcome.
- April 27, 2016: Subgroup analysis was added. Analyses on the following subgroups: Cardiovascular-, infectious-, surgical-, neurological diseases and patients aged older than 65 year. Patients will be eligible for a subgroup based on their primary diagnosis at discharge.
- May 3, 2016: The follow-up period for each patient was changed from 12 months from discharge to one date for all patients, where follow-up ends: 10 months after admission of the last patient: April 6, 2017. This is approximately equal to a median follow-up of 12 months.
- May 31, 2016: Change from 3 to 2 participating centers, which also meant a change in the design, reducing number of control periods to be equal the number of intervention periods. It was clear in the beginning of March that Nordsjælland Hospital had withdrawn from the study. The Steering Committee tried in the subsequent months to find a replacement, which was not possible. The late change on clinical[trials.gov](http://trials.gov) reflected this.
- June 6, 2016: Status of study changed because of planned stop in the inclusion period.
- April 5, 2017: Original Statistical Analysis plan was made available online.
