# Supplementary material for: Use of the prognostic biomarker suPAR in the emergency department improves risk stratification but has no effect on mortality: a cluster-randomized clinical trial (TRIAGE III)
Source: Scand J Trauma Resusc Emerg Med. 2018 Aug 28;26:69. doi: 10.1186/s13049-018-0539-5 (PMC6114851; doi:10.1186/s13049-018-0539-5)
Supplement: Supplementary file 2 — Appendix contains additional and supporting tables and figures for the manuscript. (DOCX 256 kb) [file 13049_2018_539_MOESM2_ESM.docx]

**Appendix**

**TRIAGE III trial**

All documents included are from the TRIAGE III trial.

**Table of contents**

[List of Steering Committee members 2](#_Toc515348470)

[List of investigators 3](#_Toc515348471)

[Inclusion and exclusion criteria for patients 4](#_Toc515348472)

[Additional description of statistics 5](#_Toc515348473)

[Appendix Figures 6](#_Toc515348474)

[Appendix Tables 9](#_Toc515348475)

# List of Steering Committee members

Kasper Iversen, M.D., D.M.Sc.^1^

Jesper Eugen-Olsen, Ph.D.^2^

Lars Simon Rasmussen, M.D., D.M.Sc.^3^

Lars Køber, M.D., D.M.Sc.^4^

Erik Kjøller, M.D., D.M.Sc.^1^

Birgitte Nybo Jensen, M.D., Ph.D.^5^

Lisbet Ravn, M.D.^6^

**Chief Statistician:**

Theis Lange, Ph.D.^7,8^

**Data Manager:**

Thomas Kallemose, MSc.^2^

**Affiliations:**

1Department of Cardiology, Herlev and Gentofte Hospital, University of Copenhagen, Herlev Ringvej 75, 2730 Herlev, Denmark. 2Clinical Research Centre, Hvidovre and Amager Hospital, University of Copenhagen, Kettegård Alle 30, 2650 Hvidovre, Denmark. 3Department of Anaesthesia, Rigshospitalet, University of Copenhagen, Blegdamsvej 9, 2100 Copenhagen, Denmark. 4Department of Cardiology, Rigshospitalet, University of Copenhagen, Blegdamsvej 9, 2100 Copenhagen, Denmark. 5Department of Emergency Medicine, Bispebjerg Hospital, University of Copenhagen, Bispebjerg Bakke 23, 2400 Copenhagen, Denmark. 6Department of Emergency Medicine, Herlev Hospital, University of Copenhagen, Herlev Ringvej 75, 2730 Herlev, Denmark. ^7^Department of Public Health, University of Copenhagen, Section of biostatistics, Øster Farimagsgade 5, 1014, Copenhagen, Denmark, and Center for Statistical Science, Peking University, No. 5 Yiheyuan Road Haidian District, Beijing, 100871, China.

.

# List of investigators

*Herlev and Gentofte Hospital, University of Copenhagen*

Martin Schultz Department of Cardiology and Internal Medicine

Malene H. Andersen Department of Cardiology

Sarah Holle Department of Cardiology

Morten Alstrup Department of Cardiology

Andreas Sandø Department of Cardiology

Thomas Høi-Hansen Department of Cardiology

Kasper Iversen Department of Cardiology

Morten Lind Department of Emergency medicine

Lisbet Ravn Department of Emergency medicine

*Bispebjerg Hospital, University of Copenhagen*

Birgitte Nybo Jensen Department of Emergency medicine

*Hvidovre and Amager Hospital, University of Copenhagen*

Jeppe Meyer Clinical Research Center

Peter B S Andersen Clinical Research Center

Line Jee Hartmann Rasmussen Clinical Research Center

Thomas Kallemose Clinical Research Center

Jesper Eugen-Olsen Clinical Research Center

*Rigshospitalet, University of Copenhagen*

Jakob S. Stefansson Department of Anaesthesia

Alexander C. Falkentoft Department of Cardiology

Lars Køber Department of Cardiology

Lars Simon Rasmussen Department of Anaesthesia

# Inclusion and exclusion criteria for patients

**Inclusion criteria**

1. ≥16 years at admittance.
2. Acute arrival at the emergency department within the inclusion period.
3. Have routine blood tests analyzed, including hemoglobin, C-reactive protein, and creatinine, within 6 hours of admission to the emergency department.
4. As the results of the blood tests can take up to two hours to provide, the doctors cannot change outcome based on biomarker levels until those data are provided. Hence, inclusion time will be calculated as time for blood sampling + 2 hours.

**Exclusion criteria**

1. Arrival at the gynecological-, obstetric-, and pediatric department.

# Additional description of statistics

**Definitions of specific secondary outcomes**

In-hospital admission is defined as admissions at the ED longer than 24 hours. Some patients remained in the ED for treatment and subsequent discharge, the majority of patients was transferred to stationary wards at the participating hospitals.

Admissions to the medical wards is defined as all ED visits allocated to the medical area of the ED and all transfer from this or other ED area to a stationary medical ward. Admissions to all internal medicine wards (excluding cardiology) and at medical areas of the ED were combined, which is in accordance with the data from the Danish National Patient Registry.

# Appendix Figures

**Appendix Figure 1. Survival of the TRIAGE III population stratified by suPAR quartiles.**

**
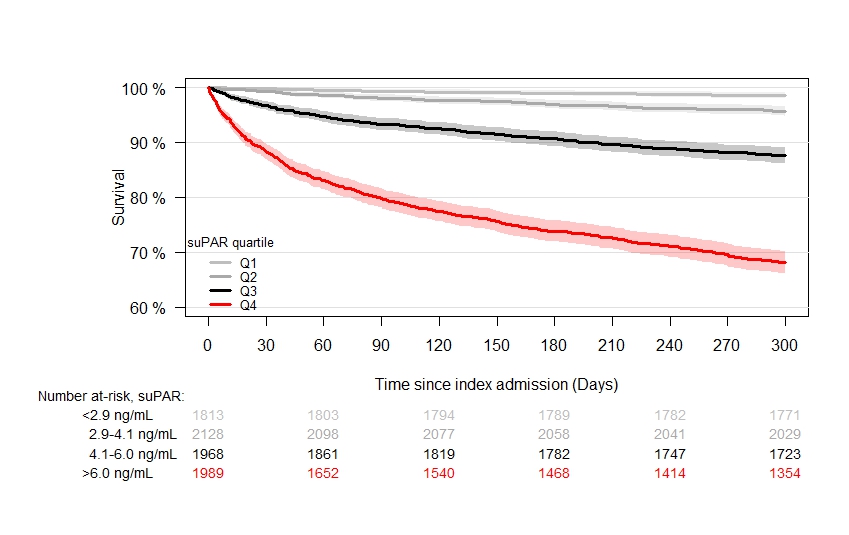
**

**Appendix Figure 1.** Kaplan-Meier plot displaying survival of patients acutely admitted at two emergency departments stratified by quartiles of soluble urokinase plasminogen activator receptor (suPAR). Log-rank test: P<0.001.

**Appendix Figure 2. Result of survey among doctors participating in the TRIAGE III trial.** **
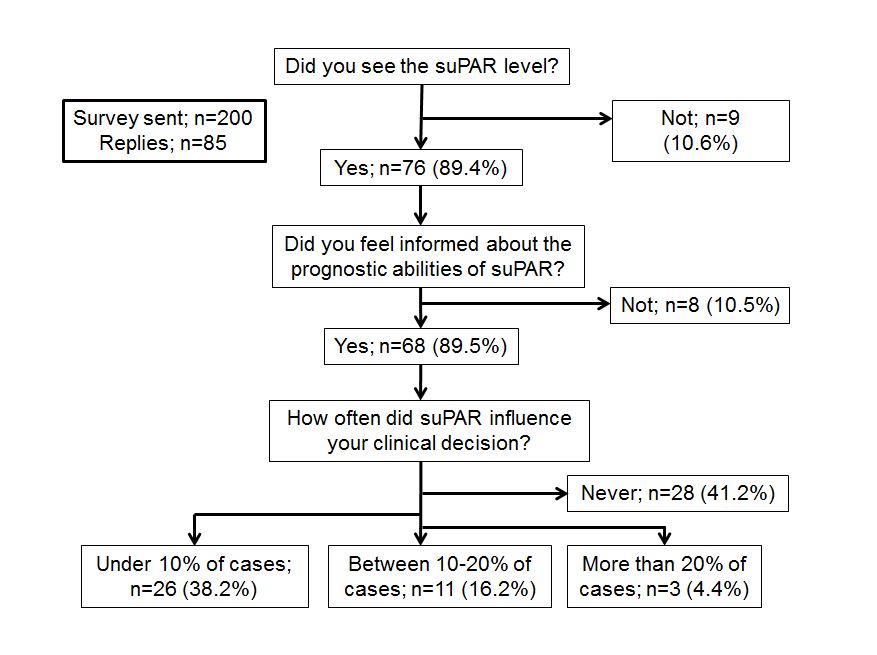
**

**Appendix Figure 2.** Flow chart showing the replies from the web-based anonymous survey of 200 randomly selected emergency department doctors in the TRIAGE III trial, in which patients who were acutely admitted to the emergency department were allocated to measurement of soluble urokinase plasminogen activator receptor (suPAR) as a prognostic marker or to control (no suPAR measurement).

# Appendix Tables

| Appendix Table 1. Characteristics of acutely admitted patients at the index admission, stratified by cluster. | | | | |
| --- | --- | --- | --- | --- |
| Hospital | **Herlev hospital** | | **Bispebjerg hospital** | |
| Patient Characteristic  No. of patients | **Intervention**  **(N = 5,449)** | **Control**  **(N=4,332)** | **Intervention**  **(N=3,451**) | **Control**  **(N=3,569)** |
| Female sex, no. (%) | 2,913 (53.5) | 2,256 (52.1) | 1,776 (51.5) | 1,919 (53.8) |
| Age (years), mean (SD) | 60.98 (20.30) | 61.06 (20.52) | 59.45 (21.44) | 60.62 (20.90) |
| Charlson score, mean (SD) | 0.75 (1.56) | 0.71 (1.53) | 0.60 (1.28) | 0.69 (1.42) |
| Biomarkers, median (IQR) |  |  |  |  |
| Albumin (g/L) | 42 (38–44) | 41 (38–44) | 35 (31–39) | 35 (31–39) |
| Creatinine (µmol/L) | 72 (60–90) | 72 (60–89) | 80 (67–97) | 78 (65–97) |
| CRP (mg/L) | 3.0 (3.0–41.0) | 3.0 (3.0–41.0) | 7.7 (2.0–37.0) | 8.0 (2.0–42.0) |
| Hemoglobin (mmol/L) | 8.3 (7.5-9.0) | 8.3 (7.5-9.0) | 8.4 (7.6–9.1) | 8.3 (7.5–9.0) |
| suPAR (ng/ml) | 4.1 (3.0–5.9) | n.a. | 4.1 (2.9–6.1) | n.a |
| Admissions |  |  |  |  |
| Length of hospital stay (days), mean (SD) | 3.95 (6.64) | 4.11 (8.28) | 5.08 (10.29) | 5.03 (9.16) |
| Transfer to medical ward, n (%) | 1,985 (36.5) | 1,609 (37.3) | 1,752 (50.8) | 1,887 (52.9) |
| Transfer to the ICU, n (%) | 39 (0.7) | 38 (0.9) | 80 (2.3) | 65 (1.8) |
| Subgroups, diagnoses at discharged |  |  |  |  |
| Cancers, n (%) | 461 (8.5) | 350 (8.1) | 98 (2.8) | 121 (3.4) |
| Cardiovascular disease, no. (%) | 1014 (18.6) | 769 (17.8) | 904 (26.2) | 957 (26.8) |
| Infections, no. (%) | 969 (17.8) | 817 (18.9) | 707 (20.5) | 767 (21.5) |
| Neurological disease, no. (%) | 489 (9.0) | 400 (9.2) | 447 (13.0) | 464 (13.0) |
| Surgery during admission, no. (%) | 474 (8.7) | 411 (9.5) | 376 (10.5) | 402 (11.6) |
| Outcomes |  |  |  |  |
| Mortality during follow-up, no. (%) | 814 (14.9) | 611 (14.1) | 427 (12.4) | 515 (14.4) |
| New cancers during follow up, no. (%) | 538 (9.9) | 449 (10.4) | 239 (6.9) | 251 (7.0) |
| Patients discharged within 24 h, no (%) | 2,961 (54.3) | 2,356 (54.4) | 1,468 (42.3) | 1,498 (42.0) |
| Readmission within follow up, no. (%) | 1,608 (29.5) | 1,262 (29.1) | 1,036 (30.0) | 1,061 (29.7) |
| CRP: C-reactive protein, ICU: Intensive care unit, IQR: interquartile range, SD: standard deviation. | | | |  |

| Appendix Table 2. Different Cox models on all-cause mortality at end of follow-up. TRIAGE III trial. | | |
| --- | --- | --- |
| Model | **HR (95% CI)** | **P-value** |
|  |  |  |
| Intention to treat (ITT) |  |  |
| Weighted Cox, unadjusted | 0.97 (0.89–1.07) | 0.57 |
| Weighted Cox, adjusted for age, sex, hospital | 0.99 (0.91–1.09) | 0.88 |
| Weighted Cox, adjusted for age, sex, hospital, Charlson score and CRP | 1.04 (0.95–1.14) | 0.41 |
| Censoring at readmission, adjusted for age, sex, hospital, Charlson score and CRP | 1.06 (0.95–1.19) | 0.27 |
| Subgroups, ITT – weighted Cox – fully adjusted model | | |
| Bispebjerg hospital | 1.02 (0.88–1.19) | 0.76 |
| Herlev hospital | 1.05 (0.92–1.19) | 0.48 |
| Age < 65 years | 1.12 (0.86–1.46) | 0.41 |
| Age >= 65 years | 1.03 (0.93–1.14) | 0.54 |
| Cancer | 1.03 (0.87–1.22) | 0.75 |
| Cardiovascular disease | 1.06 (0.90–1.24) | 0.50 |
| Infectious disease | 0.92 (0.83–1.03) | 0.13 |
| Neurological disease | 1.08 (0.87–1.34) | 0.50 |
| Surgery during admission | 0.97 (0.75–1.27) | 0.85 |
| Per protocol |  |  |
| Weighted Cox, unadjusted | 0.98 (0.89–1.08) | 0.40 |
| Weighted Cox, adjusted for age, sex, hospital, Charlson score and CRP | 1.04 (0.94–1.15) | 0.45 |
| CI: confidence interval, CRP: C-reactive protein, fully adjusted model: adjusted for age, sex, hospital, Charlson Score and CRP, HR: hazard ratio, ITT: Intention to treat. Per protocol: Cohort with exclusion of patients without a suPAR measurement in the intervention periods and patients with an erroneous suPAR measurement in the control periods. | | |

| Appendix Table 3. Subgroup analyses of secondary outcomes in the intention to treat cohort. | | | |
| --- | --- | --- | --- |
| Subgroup | **Intervention** | **Control** | **P-value** |
| Bispebjerg hospital, no. of patients | 3,467 | 3,584 |  |
| Length of hospital stay (days), mean (SD) | 5.13 (10.36) | 5.04 (9.19) | 0.702 |
| Patients discharged from hospital within 24 hours, no. (%) | 1,470 (42.4) | 1,502 (41.9) | 0.701 |
| Readmissions within 30 days, no. (%) | 289 (8.3) | 300 (8.4) | 0.048* |
| Herlev hospital, no. of patients | 5,462 | 4,350 |  |
| Length of hospital stay (days), mean (SD) | 3.94 (6.63) | 4.11 (8.27) | 0.270 |
| Patients discharged from hospital within 24 hours, no. (%) | 2,969 (54.4) | 2,366 (54.6) | 0.924 |
| Readmissions within 30 days, no. (%) | 524 (9.6) | 323 (7.4) | <0.001* |
| Age < 65, no. of patients | 4,566 | 3,990 |  |
| Length of hospital stay (days), mean (SD) | 3.08 (8.10) | 3.20 (8.80) | 0.527 |
| Patients discharged from hospital within 24 hours, no. (%) | 2,952 (64.7) | 2,537 (63.7) | 0.327 |
| Readmissions within 30 days, no. (%) | 330 (7.2) | 258 (6.5) | 0.400 |
| Age >= 65, no. of patients | 4,334 | 3,912 |  |
| Length of hospital stay (days), mean (SD) | 5.76 (8.23) | 5.88 (8.39) | 0.507 |
| Patients discharged from hospital within 24 hours, no. (%) | 1,477 (34.1) | 1,317 (33.8) | 0.743 |
| Readmissions within 30 days, no. (%) | 502 (11.6) | 375 (9.6) | 0.028* |
| Cancer, no. of patients | 613 | 524 |  |
| Length of hospital stay (days), mean (SD) | 8.32 (12.76) | 8.96 (13.56) | 0.416 |
| Patients discharged from hospital within 24 hours, no. (%) | 135 (22.1) | 113 (21.9) | 0.969 |
| Readmissions within 30 days, no. (%) | 69 (11.3) | 41 (7.8) | 0.097 |
| Cardiovascular disease, no. of patients | 2,069 | 1,886 |  |
| Length of hospital stay (days), mean (SD) | 7.01 (11.77) | 6.83 (10.98) | 0.621 |
| Patients discharged from hospital within 24 hours, no. (%) | 591 (28.6) | 559 (29.7) | 0.482 |
| Readmissions within 30 days, no. (%) | 111 (5.4) | 110 (5.8) | 0.209 |
| Infections, no. of patients | 1,676 | 1,584 |  |
| Length of hospital stay (days), mean (SD) | 7.3 (10.32) | 7.3 (12.17) | 0.986 |
| Patients discharged from hospital within 24 hours, no. (%) | 454 (27.1) | 454 (28.7) | 0.097 |
| Readmissions within 30 days, no. (%) | 226 (13.5) | 158 (10.0) | 0.001* |
| Neurological disease, no. of patients | 1,008 | 923 |  |
| Length of hospital stay (days), mean (SD) | 8.74 (17.11) | 8.66 (14.86) | 0.912 |
| Patients discharged from hospital within 24 hours, no. (%) | 238 (23.8) | 234 (25.5) | 0.414 |
| Readmissions within 30 days, no. (%) | 45 (4.4) | 36 (3.9) | 0.710 |
| Surgery during admission, no. of patients | 978 | 871 |  |
| Length of hospital stay (days), mean (SD) | 7.10 (13.99) | 6.81 (11.58) | 0.633 |
| Patients discharged from hospital within 24 hours, no. (%) | 253 (25.9) | 255 (29.3) | 0.109 |
| Readmissions within 30 days, no. (%) | 101 (10.3) | 73 (8.4) | 0.367 |
| SD: standard deviation. | | |  |
