# Supplementary material for: Use of the prognostic biomarker suPAR in the emergency department improves risk stratification but has no effect on mortality: a cluster-randomized clinical trial (TRIAGE III)
Source: Scand J Trauma Resusc Emerg Med. 2018 Aug 28;26:69. doi: 10.1186/s13049-018-0539-5 (PMC6114851; doi:10.1186/s13049-018-0539-5)
Supplement: Supplementary file 3 — CONSORT checklist, documentation that the TRIAGE III trial follows the CONSORT criteria. (DOCX 29 kb) [file 13049_2018_539_MOESM3_ESM.docx]

**CONSORT 2010 checklist**

**Information to include when reporting a cluster randomized trial**

| Section/Topic | Item No | Standard Checklist item | Extension for cluster designs | Page No * |
| --- | --- | --- | --- | --- |
| Title and abstract | | | |  |
|  | 1a | Identification as a randomized trial in the title | Identification as a cluster randomized trial in the title | **Title page 1** |
|  | 1b | Structured summary of trial design, methods, results, and conclusions (for specific guidance see CONSORT for abstracts)^[[1]](#endnote-1),^^[[2]](#endnote-2)^ | See table 2 |  |
| Introduction | | | |  |
| Background and objectives | 2a | Scientific background and explanation of rationale | Rationale for using a cluster design | **Methods p. 5** |
|  | 2b | Specific objectives or hypotheses | Whether objectives pertain to the cluster level, the individual participant level or both | **Methods p. 5** |
| Methods | | | |  |
| Trial design | 3a | Description of trial design (such as parallel, factorial) including allocation ratio | Definition of cluster and description of how the design features apply to the clusters | **Methods p. 5** |
|  | 3b | Important changes to methods after trial commencement (such as eligibility criteria), with reasons |  | **Results p. 9 Figure 1** |
| Participants | 4a | Eligibility criteria for participants | Eligibility criteria for clusters | **Methods p. 5** |
|  | 4b | Settings and locations where the data were collected |  | **Methods p. 5** |
| Interventions | 5 | The interventions for each group with sufficient details to allow replication, including how and when they were actually administered | Whether interventions pertain to the cluster level, the individual participant level or both | **Methods p. 5-6** |
| Outcomes | 6a | Completely defined pre-specified primary and secondary outcome measures, including how and when they were assessed | Whether outcome measures pertain to the cluster level, the individual participant level or both | **Methods p. 5** |
|  | 6b | Any changes to trial outcomes after the trial commenced, with reasons |  | **Supplementary material: protocol and SAP p. 45-47** |
| Sample size | 7a | How sample size was determined | Method of calculation, number of clusters(s) (and whether equal or unequal cluster sizes are assumed), cluster size, a coefficient of intracluster correlation (ICC or *k*), and an indication of its uncertainty | **Methods p. 7** |
|  | 7b | When applicable, explanation of any interim analyses and stopping guidelines |  | **Not relevant** |
| Randomisation: | | | |  |
| Sequence generation | 8a | Method used to generate the random allocation sequence |  | **Methods p. 5** |
|  | 8b | Type of randomization; details of any restriction (such as blocking and block size) | Details of stratification or matching if used | **Methods p. 5** |
| Allocation concealment mechanism | 9 | Mechanism used to implement the random allocation sequence (such as sequentially numbered containers), describing any steps taken to conceal the sequence until interventions were assigned | Specification that allocation was based on clusters rather than individuals and whether allocation concealment (if any) was at the cluster level, the individual participant level or both | **Methods p. 5** |
| Implementation | 10 | Who generated the random allocation sequence, who enrolled participants, and who assigned participants to interventions | Replace by 10a, 10b and 10c |  |
|  | 10a |  | Who generated the random allocation sequence, who enrolled clusters, and who assigned clusters to interventions | **Methods p. 5** |
|  | 10b |  | Mechanism by which individual participants were included in clusters for the purposes of the trial (such as complete enumeration, random sampling) | **Methods p. 5** |
|  | 10c |  | From whom consent was sought (representatives of the cluster, or individual cluster members, or both), and whether consent was sought before or after randomization | **Ethics p. 15** |
|  |  |  |  |  |
| Blinding | 11a | If done, who was blinded after assignment to interventions (for example, participants, care providers, those assessing outcomes) and how |  | **Not relevant** |
|  | 11b | If relevant, description of the similarity of interventions |  | **Not relevant** |
| Statistical methods | 12a | Statistical methods used to compare groups for primary and secondary outcomes | How clustering was taken into account | **Methods p. 8** |
|  | 12b | Methods for additional analyses, such as subgroup analyses and adjusted analyses |  | **Methods p. 8** |
| Results | | | |  |
| Participant flow (a diagram is strongly recommended) | 13a | For each group, the numbers of participants who were randomly assigned, received intended treatment, and were analyzed for the primary outcome | For each group, the numbers of clusters that were randomly assigned, received intended treatment, and were analyzed for the primary outcome | **Results p. 10 and figure 1** |
|  | 13b | For each group, losses and exclusions after randomization, together with reasons | For each group, losses and exclusions for both clusters and individual cluster members | **Results p. 9and figure 1** |
| Recruitment | 14a | Dates defining the periods of recruitment and follow-up |  | **Results p. 9** |
|  | 14b | Why the trial ended or was stopped |  | **Results p. 9** |
| Baseline data | 15 | A table showing baseline demographic and clinical characteristics for each group | Baseline characteristics for the individual and cluster levels as applicable for each group | **Table 1 and Appendix S1** |
| Numbers analyzed | 16 | For each group, number of participants (denominator) included in each analysis and whether the analysis was by original assigned groups | For each group, number of clusters included in each analysis | **Results p. 9 and figure 1** |
| Outcomes and estimation | 17a | For each primary and secondary outcome, results for each group, and the estimated effect size and its precision (such as 95% confidence interval) | Results at the individual or cluster level as applicable and a coefficient of intracluster correlation (ICC or k) for each primary outcome | **Results p. 10** |
|  | 17b | For binary outcomes, presentation of both absolute and relative effect sizes is recommended |  | **Results p.10** |
| Ancillary analyses | 18 | Results of any other analyses performed, including subgroup analyses and adjusted analyses, distinguishing pre-specified from exploratory |  | **Results p. 9-11 and Appendix** |
| Harms | 19 | All important harms or unintended effects in each group (for specific guidance see CONSORT for harms^[[3]](#endnote-3)^) |  | **Not relevant** |
| Discussion | | | |  |
| Limitations | 20 | Trial limitations, addressing sources of potential bias, imprecision, and, if relevant, multiplicity of analyses |  | **Discussion p. 13-14** |
| Generalizability | 21 | Generalizability (external validity, applicability) of the trial findings | Generalizability to clusters and/or individual participants (as relevant) | **Discussion p. 12-13** |
| Interpretation | 22 | Interpretation consistent with results, balancing benefits and harms, and considering other relevant evidence |  | **Discussion p. 13** |
| Other information | | |  |  |
| Registration | 23 | Registration number and name of trial registry |  | **Registration p. 3** |
| Protocol | 24 | Where the full trial protocol can be accessed, if available |  | **Methods p. 5** |
| Funding | 25 | Sources of funding and other support (such as supply of drugs), role of funders |  | **Page 16** |

1. Hopewell S, Clarke M, Moher D, Wager E, Middleton P, Altman DG, et al. CONSORT for reporting randomized trials in journal and conference abstracts. *Lancet* 2008, 371:281-283 [↑](#endnote-ref-1)
2. Hopewell S, Clarke M, Moher D, Wager E, Middleton P, Altman DG at al (2008) CONSORT for reporting randomized controlled trials in journal and conference abstracts: explanation and elaboration. *PLoS Med* 5(1): e20 [↑](#endnote-ref-2)
3. Ioannidis JP, Evans SJ, Gotzsche PC, O'Neill RT, Altman DG, Schulz K, Moher D. Better reporting of harms in randomized trials: an extension of the CONSORT statement. *Ann Intern Med* 2004; 141(10):781-788. [↑](#endnote-ref-3)
